# Supplementary material for: A multi-tiered mechanical mechanism shapes the early neural plate
Source: Nat Commun. 2025 Jul 4;16:6187. doi: 10.1038/s41467-025-61303-1 (PMC12227548; doi:10.1038/s41467-025-61303-1)
Supplement: Supplementary file 1 — Supplementary Information [file 41467_2025_61303_MOESM1_ESM.pdf]

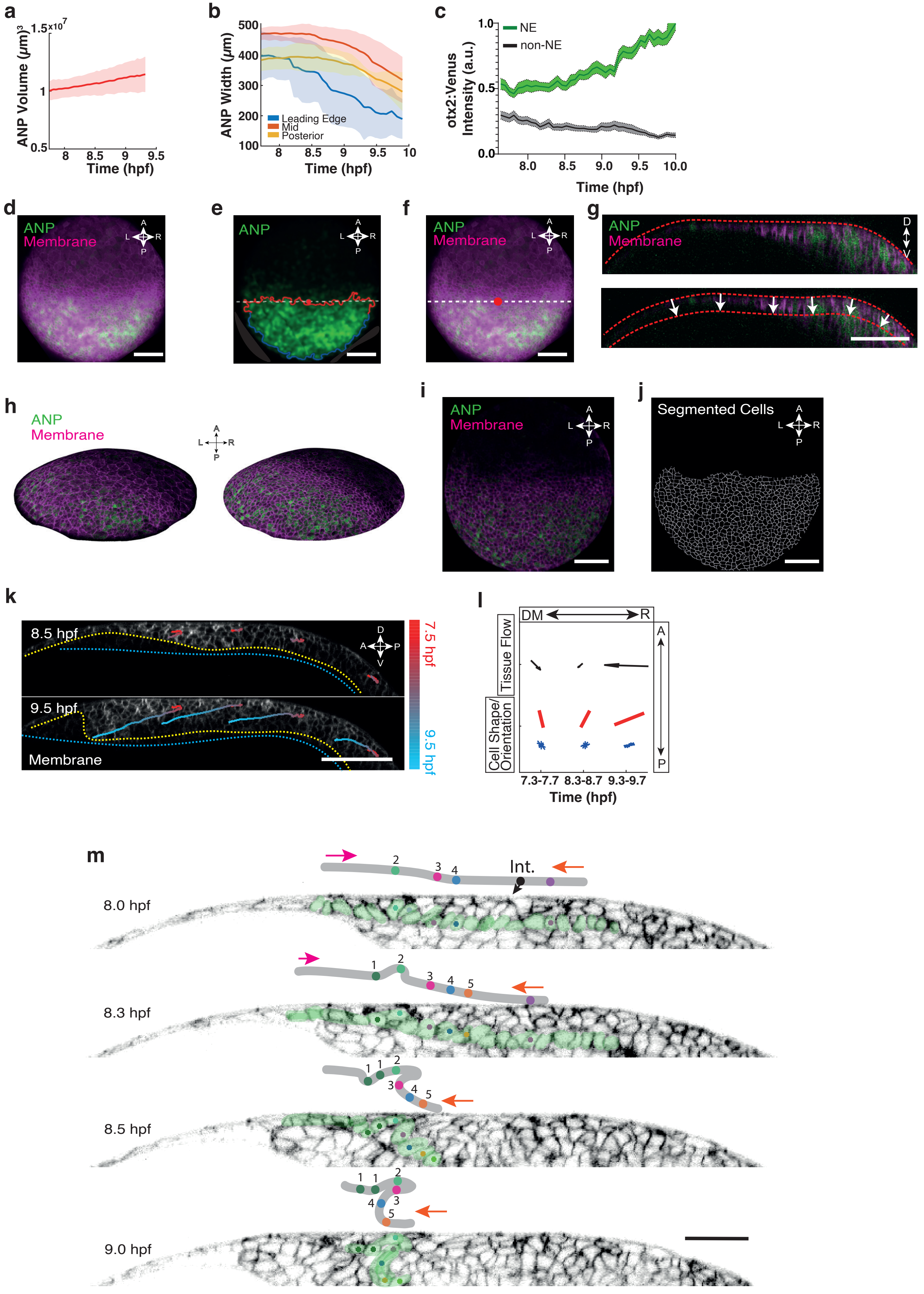
a
ANP Volume (μm)³
1.5x10⁷
1
0.5
8 8.5 9 9.5
Time (hpf)
b
ANP Width (μm)
500 400 300 200 100
Leading Edge
Mid
Posterior
8 8.5 9 9.5 10
Time (hpf)
c
otx2:Venus Intensity (a.u.)
NE
non-NE
1.0 0.5 0.0
8.0 8.5 9.0 9.5 10.0
Time (hpf)
d
ANP
Membrane
e
ANP
f
ANP
Membrane
g
ANP
Membrane
D
V
h
ANP
Membrane
i
ANP
Membrane
j
Segmented Cells
k
8.5 hpf
9.5 hpf
Membrane
7.5 hpf
9.5 hpf
l
DM
R
A
P
Tissue Flow
Cell Shape/ Orientation
7.3-7.7 8.3-8.7 9.3-9.7
Time (hpf)
m
Int.
8.0 hpf
8.3 hpf
8.5 hpf
9.0 hpf

Supplementary Figure 1: **Image processing and analysis of anterior neural plate movements and shape changes during gastrulation.**

a) Anterior neural plate (ANP) tissue volume in wild type (WT) embryos during gastrulation. **b)** ANP width at different locations from anterior to posterior edge in WT embryos during gastrulation. **c)** Normalised otx2:Venus expression in neuroectoderm (NE) (green) vs non-NE (grey) cells during gastrulation (10 NE, 10 non-NE cells per embryo). **d)** Mean intensity projection of membrane marker (mRFP) and ANP tissue marker (otx2:Venus) in WT embryos. Animal pole view; anterior (A), posterior (P), left (L), right (R). **e)** Identifying the anterior leading edge of the otx2:Venus region as the approximately linear portion (red) of the contour surrounding the region, with the origin (red circle) placed at the centre of mass projected on the linear approximation (white dashed line) of the leading edge. **f)** The projection in (d) indicating the ANP leading edge (white dashed line) and the AP axis/leading edge intersection (red dot). Membrane (mRFP, magenta) and ANP (otx2:Venus, green) labelled. **g)** Segmented surface (red dashes) translated inwards along the surface normal (white arrows), allowing the mapping of points on the interior into the translated surface (inner red line). Sagittal sections, anterior left, dorsal (D) up, ventral (V) down. **h)** Surfaces mapped from (d) at two locations, moving inwards from left to right. **i)** Surface fluorescence values from (h) mapped onto the plane. **j)** Segmented cells from surface projection shown in (i). **k)** Sagittal confocal images with representative cells within the ANP of WT embryos tracked during gastrulation. Cell membranes (mRFP, white) labelled. Yellow dashes: NE/ME border. Blue dashes: ME/yolk interface. Cell tracks coloured from red (7.5 hpf) to blue (9.5hpf). **l)** Orientation of tissue flow versus cell orientation and shape from the right half (R) of the ANP tissue towards the dorsal

midline (DM). Arrows indicate average direction of tissue flows over 20 minutes either side of the indicated time point. Red lines: average cell shape/orientation. Blue lines: histogram of individual cell orientations (15 cells, 3 embryos). **m)** Representative confocal image series and illustrations showing tracked neuroectoderm cell positions over time within a single ANP tissue layer (green pseudocolour). Coloured dots indicate single cells. Purple arrow, posterior and orange arrow, anterior movements. Internalisation (Int) start point indicated. Orientation as in k. All data are presented as the mean ± SEM of 3 embryos, unless otherwise stated. Scale bars: 100 μm (d, e, f, g, i, j, k); 50 μm (m).

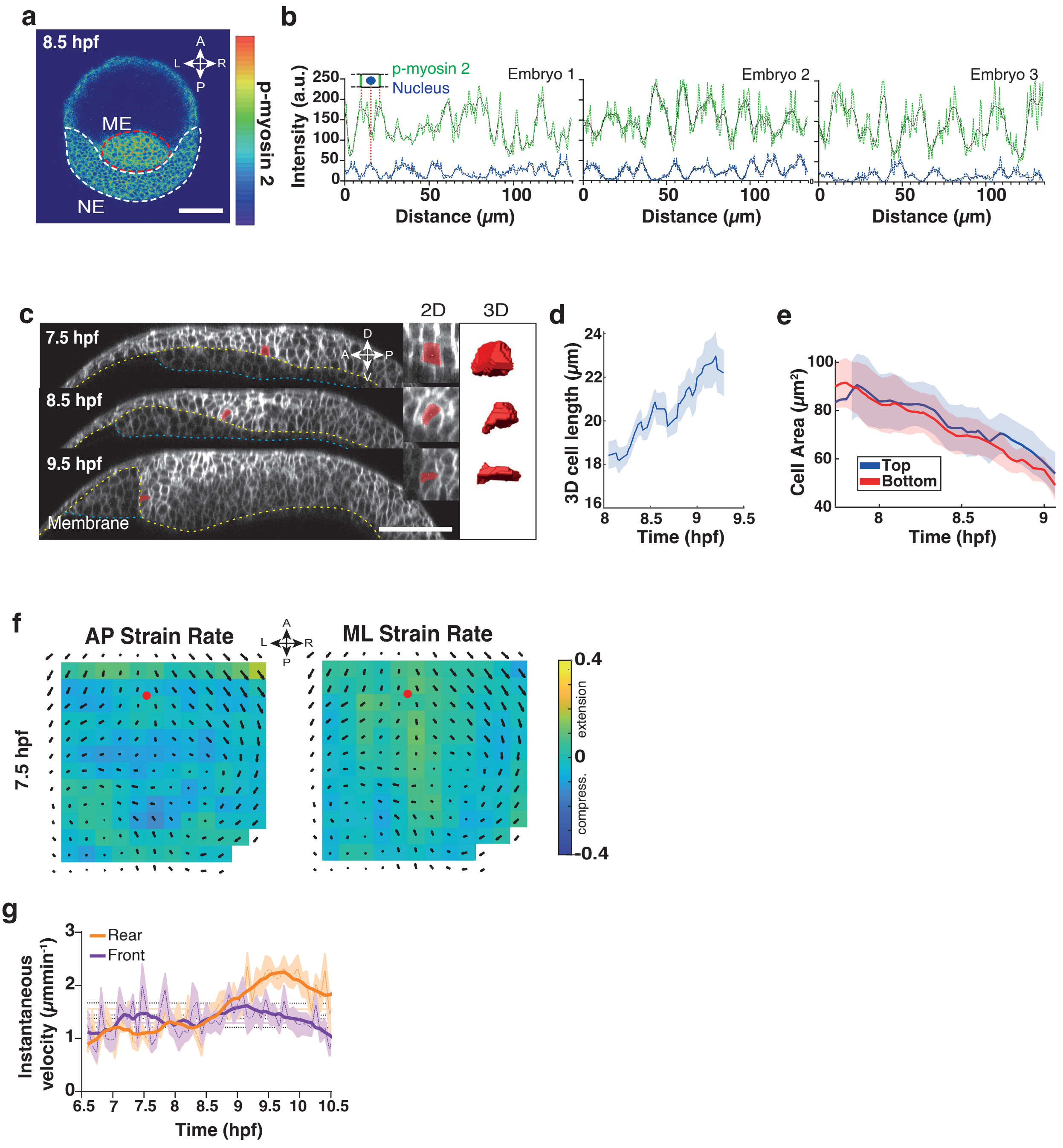
a
8.5 hpf
A
L
R
P
ME
NE
p-myosin 2
b
p-myosin 2
Nucleus
Intensity (a.u.)
Embryo 1
Embryo 2
Embryo 3
Distance (μm)
c
7.5 hpf
8.5 hpf
9.5 hpf
2D
3D
D
A
P
V
Membrane
d
3D cell length (μm)
Time (hpf)
e
Cell Area (μm²)
Top
Bottom
Time (hpf)
f
AP Strain Rate
ML Strain Rate
7.5 hpf
extension
compress.
0.4
0
-0.4
g
Rear
Front
Instantaneous velocity (μmmin⁻¹)
Time (hpf)

Supplementary Figure 2: Heterotypic tissue interactions drive neuroectoderm internalisation and multilayer tissue folding.

a) Whole mount p-myosin 2 stained multiphoton image of wild type (WT) embryo. White area demarcates neuroectoderm (NE) cells in the anterior neural plate (ANP), red area indicates mesendoderm (ME). **b)** Normalised and smoothed individual line scans of H2A-BFP (blue) and p-myosin 2 (green) expression in NE cells across the ML direction centred on the dorsal midline during internalisation analysed from individual embryos (n = 3). Schematic illustrates p-myosin peaks at cell-cell contacts and H2A-BFP peaks at the cell nucleus. **c)** Sagittal confocal images representing the ANP in WT embryos during gastrulation. Dorsal up, ventral down, anterior left, posterior right. Cell membranes (mRFP, white) labelled. Yellow dashes: NE ventral and anterior border to ME. Blue dashes: ME/yolk interface. Representative internalising cell (red) illustrates shape changes in 2D and 3D over time. **d)** Changes in ME cell length during internalisation based on 3D reconstruction measurements. (25 cells, 3 embryos) **e)** Top/dorsal (blue) and bottom/ventral (red) surface area of cells around internalisation (30 cells, 3 embryos). **f)** Time-averaged ANP domain strain rates along the anterior-posterior (AP) and mediolateral (ML) axes of WT embryos (7.5 hpf). Minimum, green (0); maximum stretching, yellow; maximum compression, dark blue. Black arrows: time-averaged tissue flows. Red dot: AP axis/ANP leading edge intersection. **g)** Smoothed instantaneous velocity of individual cells in the front (purple) and rear (orange) of the ME during gastrulation (5 front and 5 rear cells per embryo). All data are presented as the mean ± SEM of 3 embryos, except otherwise stated. Scale bars: 100 μm.

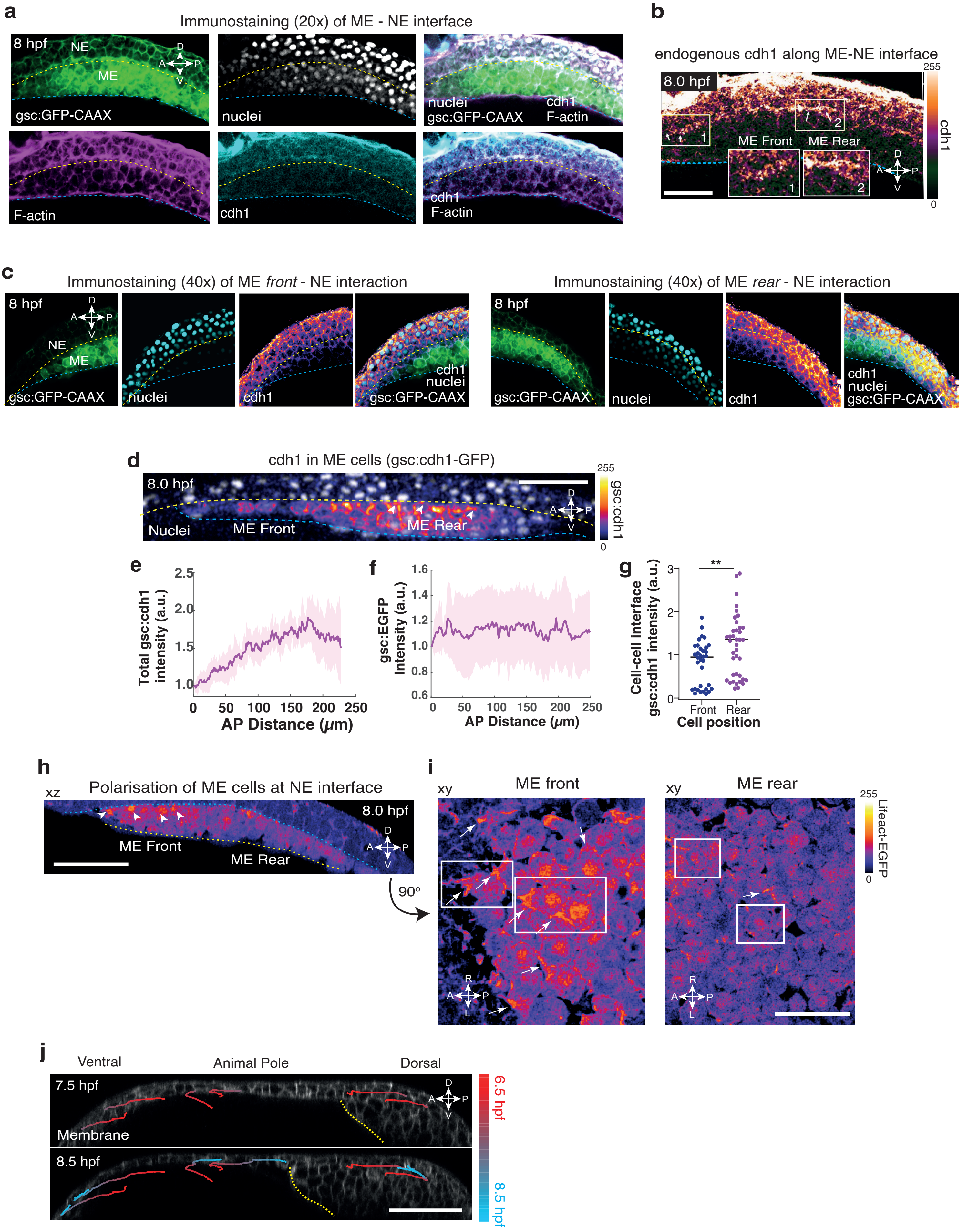
a
Immunostaining (20x) of ME - NE interface
8 hpf
NE
ME
gsc:GFP-CAAX
nuclei
nuclei
gsc:GFP-CAAX
cdh1
F-actin
F-actin
cdh1
cdh1
F-actin
b
endogenous cdh1 along ME-NE interface
8.0 hpf
ME Front
ME Rear
255
cdh1
0
c
Immunostaining (40x) of ME front - NE interaction
Immunostaining (40x) of ME rear - NE interaction
8 hpf
NE
ME
gsc:GFP-CAAX
nuclei
cdh1
cdh1
nuclei
gsc:GFP-CAAX
d
cdh1 in ME cells (gsc:cdh1-GFP)
8.0 hpf
Nuclei
ME Front
ME Rear
gsc:cdh1
e
Total gsc:cdh1 intensity (a.u.)
AP Distance (μm)
f
gsc:EGFP Intensity (a.u.)
AP Distance (μm)
g
Cell-cell interface gsc:cdh1 intensity (a.u.)
Front
Rear
Cell position
h
xz
Polarisation of ME cells at NE interface
8.0 hpf
ME Front
ME Rear
90°
i
xy
ME front
ME rear
Lifeact-EGFP
j
Ventral
Animal Pole
Dorsal
7.5 hpf
Membrane
8.5 hpf
6.5 hpf
8.5 hpf

Supplementary Figure 3. Differential mesendoderm behaviour regulates neuroectoderm cell internalisation.

a) Sagittal confocal images (20x objective) of *Tg(gsc:GFP-CAAX)* embryo with nuclei (H2A-BFP, grey) and F-actin (Phalloidin, magenta) and stained for cdh1 (cyan) showing the mesendoderm (ME) – neuroectoderm (NE) interface at 8 hours post fertilisation (hpf). Yellow dashes: NE border to ME (slight offset). Blue dashes: ME/yolk interface (slight offset ventral). Dorsal (D), ventral (V), anterior (A), posterior (P). Single channels and merged. **b)** Sagittal confocal imaging (20x objective) of interface between front (anterior) and rear (posterior) ME and overlying neuroectoderm (NE) in WT embryo (8 hpf) stained for endogenous cdh1 (Blue_Orange_ICB LUT; white maximum, black minimum). White arrows indicate contacts between ME and NE cells. Blue dashes demark the ME-yolk interface. Magnified areas of front (box 1) and rear (box 2) ME below showing single cell-cell contacts between ME and adjacent NE cells. Orientation as (a). **(c)** Sagittal multiphoton imaging (40x objective) highlighting front (left panels) and rear (right panels) ME and NE in WT embryo (8 hpf) stained for endogenous cdh1 (Fire LUT; white maximum, black minimum), nuclei (cyan) and gsc:GFP-CAAX expression. Yellow dashes demark ME-NE interface, blue dashes demark the ME-yolk interface (slight offset dorsal). Orientation as (a). **d)** Sagittal multiphoton image of *Tg(gsc:cdh1-EGFP)* embryo with nuclear (grey LUT) and E-cadherin (Fire LUT; white maximum, black minimum) in the ME. Orientation and labels as in (a). **e)** Distribution of normalised E-cadherin intensity under *gsc* promoter (gsc:cdh1) in ME cells adjacent to NE interface measured along the front-rear (AP) axis (1 = leading edge). **f)** Distribution of normalised gsc:EGFP intensity in ME cells adjacent to NE

interface measured along the AP axis (1 = leading edge). **g)** Quantification of gsc:cdh1-EGFP localisation at the cell-cell interface between ME and overlying NE cells in the front (blue) and rear (purple) of the collective. (30 cells, 3 embryos). Two tailed t-test (p=0.004). **h)** Sagittal multiphoton image (8 hpf) of *Tg(actb1:lifeact-EGFP)* embryo showing F-actin localisation (Fire LUT colour code; white maximum, black minimum). Orientation as in (a). White arrowheads indicate F-actin rich protrusions in ME front. **i)** Multiphoton images (*xy* sections) of (h), showing cells at the front and rear of the ME. Arrows indicate cell protrusions. Boxes indicate magnified areas used in Fig 2i. **j)** Sagittal confocal images with time-resolved NE cell tracks within the ANP around the animal pole in WT embryos. Cell membranes (mRFP, grey LUT) labelled. Yellow dashes: ME leading edge. Tracks coloured red (6.5 hpf) to blue (8.5 hpf). All data are presented as the mean ± SEM of 3 embryos, except otherwise stated. Scale bars: 100 μm.

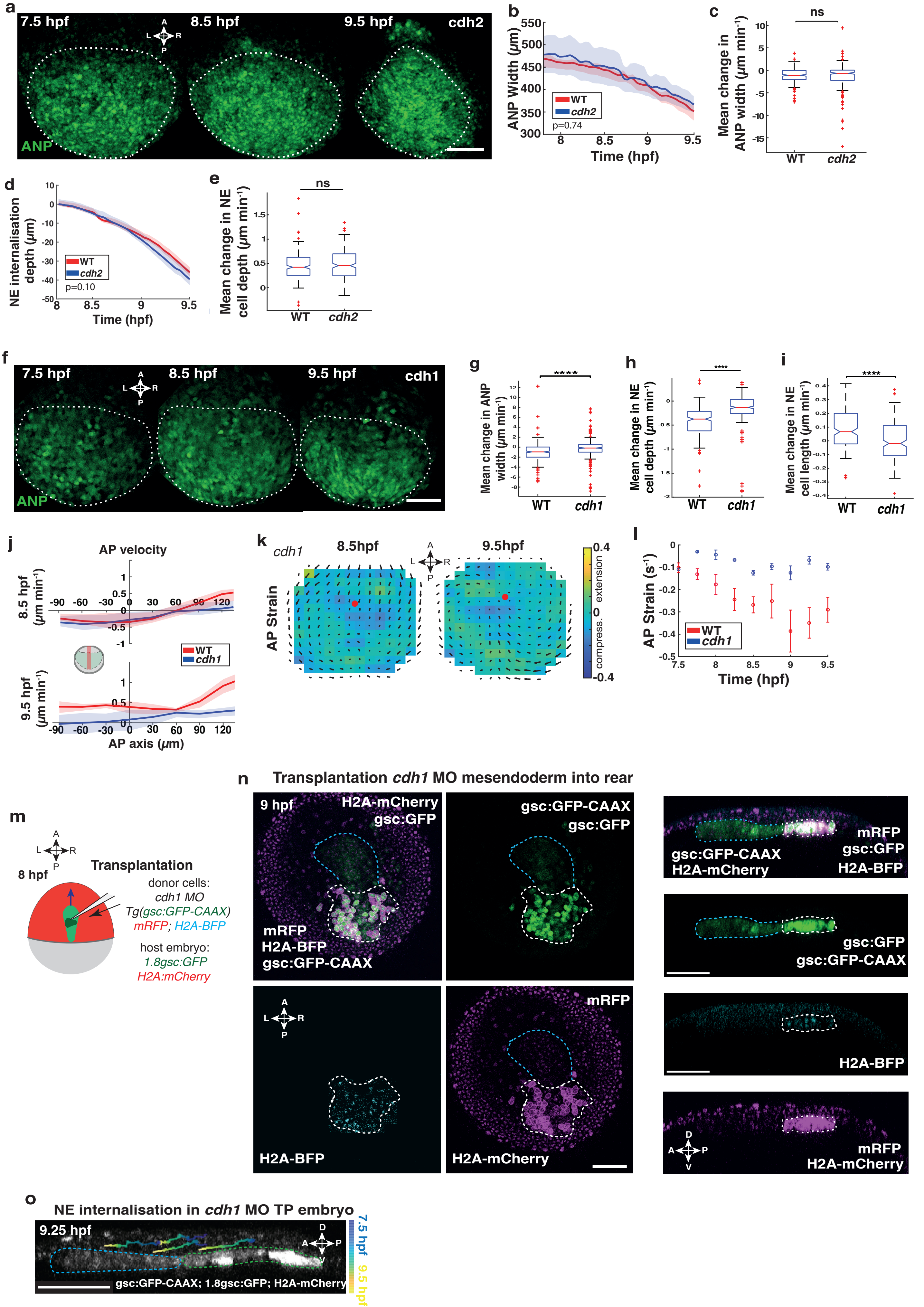

a
7.5 hpf
8.5 hpf
9.5 hpf
cdh2
ANP
b
ANP Width (µm)
WT
cdh2
p=0.74
Time (hpf)
c
ns
Mean change in ANP width (µm min⁻¹)
WT
cdh2
d
NE internalisation depth (µm)
WT
cdh2
p=0.10
Time (hpf)
e
ns
Mean change in NE cell depth (µm min⁻¹)
WT
cdh2
f
7.5 hpf
8.5 hpf
9.5 hpf
cdh1
ANP
g
****
Mean change in ANP width (µm min⁻¹)
WT
cdh1
h
****
Mean change in NE cell depth (µm min⁻¹)
WT
cdh1
i
****
Mean change in NE cell length (µm min⁻¹)
WT
cdh1
j
AP velocity
8.5 hpf (µm min⁻¹)
9.5 hpf (µm min⁻¹)
WT
cdh1
AP axis (µm)
k
cdh1
8.5hpf
9.5hpf
AP Strain
extension
compress.
l
AP Strain (s⁻¹)
WT
cdh1
Time (hpf)
m
8 hpf
Transplantation
donor cells:
cdh1 MO
Tg(gsc:GFP-CAAX)
mRFP; H2A-BFP
host embryo:
1.8gsc:GFP
H2A:mCherry
n
Transplantation cdh1 MO mesendoderm into rear
9 hpf
H2A-mCherry
gsc:GFP
mRFP
H2A-BFP
gsc:GFP-CAAX
gsc:GFP-CAAX
gsc:GFP
H2A-BFP
H2A-mCherry
mRFP
o
NE internalisation in cdh1 MO TP embryo
9.25 hpf
gsc:GFP-CAAX; 1.8gsc:GFP; H2A-mCherry
7.5 hpf
9.5 hpf

Supplementary Figure 4. E-cadherin-mediated inter-tissue interaction is required for neuroectoderm internalisation.

a) Confocal images of neuroectoderm (NE) cells (maximum projections) in the anterior neural plate (ANP) of *Tg(otx2:Venus) cadherin 2 (cdh2)* morphant embryos during gastrulation. White dashes: ANP. **b)** ANP width in wild type (WT, red) and *cdh2* morphants (blue) during gastrulation. One-way Kruskal-Wallis test (p=0.74). **c)** Mean ANP width change per minute in WT and *cdh2* morphants. One-way Kruskal-Wallis test (p=0.74). **d)** NE internalisation depth in WT and *cdh2* morphants during gastrulation (30 cells from 3 embryos). One-way Kruskal-Wallis test (p=0.10). **e)** Mean change in NE depth per minute in late gastrulation in WT and *cdh2* morphants (30 cells, 3 embryos). One-way Kruskal-Wallis test (p=0.10). **f)** Confocal images of NE cells (maximum projection) in the ANP of *Tg(otx2:Venus) cadherin-1 (cdh1)* morphant embryos during gastrulation. White dashes: ANP. **g)** Mean change in ANP width per minute in WT and *cdh1* mutants. One-way Kruskal-Wallis test ($p=1.5e^{-6}$). **h)** Mean change in ANP NE depth per minute in WT and *cdh1* morphants (30 cells, 3 embryos). One-way Kruskal-Wallis test ($p=1.3e^{-19}$). **i)** Mean change in NE cell length during internalisation in WT and *cdh1* mutants (30 cells, 3 embryos). One-way Kruskal-Wallis test ($p=3e^{-6}$). **j)** Cell velocities along the anterior-posterior (AP) axis of WT and *cdh1 mutants* at 8.5 and 9.5 hpf. Y-axis: cell velocity; X-axis (µm): 0 marks the dorsal midline. **k)** Time-averaged ANP domain strain rates along the anterior-posterior (AP) axis of *cdh1* morphants. Strain rate colour-coded minimum, green (0); maximum stretching, yellow; maximum compression, dark blue. Black arrows: average tissue flows. Red dot, AP axis/ANP leading edge intersection. **l)** Maximum absolute strain rates along the ANP midline along the AP axis of WT and *cdh1* morphants during

gastrulation. Negative values indicate compression. **m)** Transplantation schematic with labels used. *Cdh1* morpholino (MO) expressing ME cells (dark green) transplanted into the rear ME (light green) of a host embryo before internalisation at 8 hpf. **n)** Multiphoton single and merged channel images (left panels: animal pole (xy) and right panels: lateral view (zy) sections) showing host *Tg(gsc:GFP-CAAX)* embryo expressing nuclei (H2A-mCherry) marker and transplanted ME cells labelled with 1.8gsc:GFP, membrane (mRFP) and nuclei (H2A-BFP) markers. **o)** Representative sagittal multiphoton image showing colour coded tracked NE cells (from 7.5 hpf, blue to 9.5 hpf, yellow) within the ANP and transplanted *cdh1* MO cells (green dashed line) in the rear of a WT ME collective. Host embryos expressing cell nuclei (H2A:mCherry) and 1.8gsc:GFP in endogenous ME cells (grey LUT, blue dashes) and transplanted ME cells expressing gsc:EGFP-CAAX (grey LUT, green dashes). All data are presented as the mean ± SEM of 3 embryos, except otherwise stated. For box plots, central line indicates the median, and the bottom and top edges of the box indicate the 25th and 75th percentiles, respectively. The whiskers extend to the most extreme data points not considered outliers, and the outliers are plotted individually using the + marker symbol. Scale bars: 100 μm.

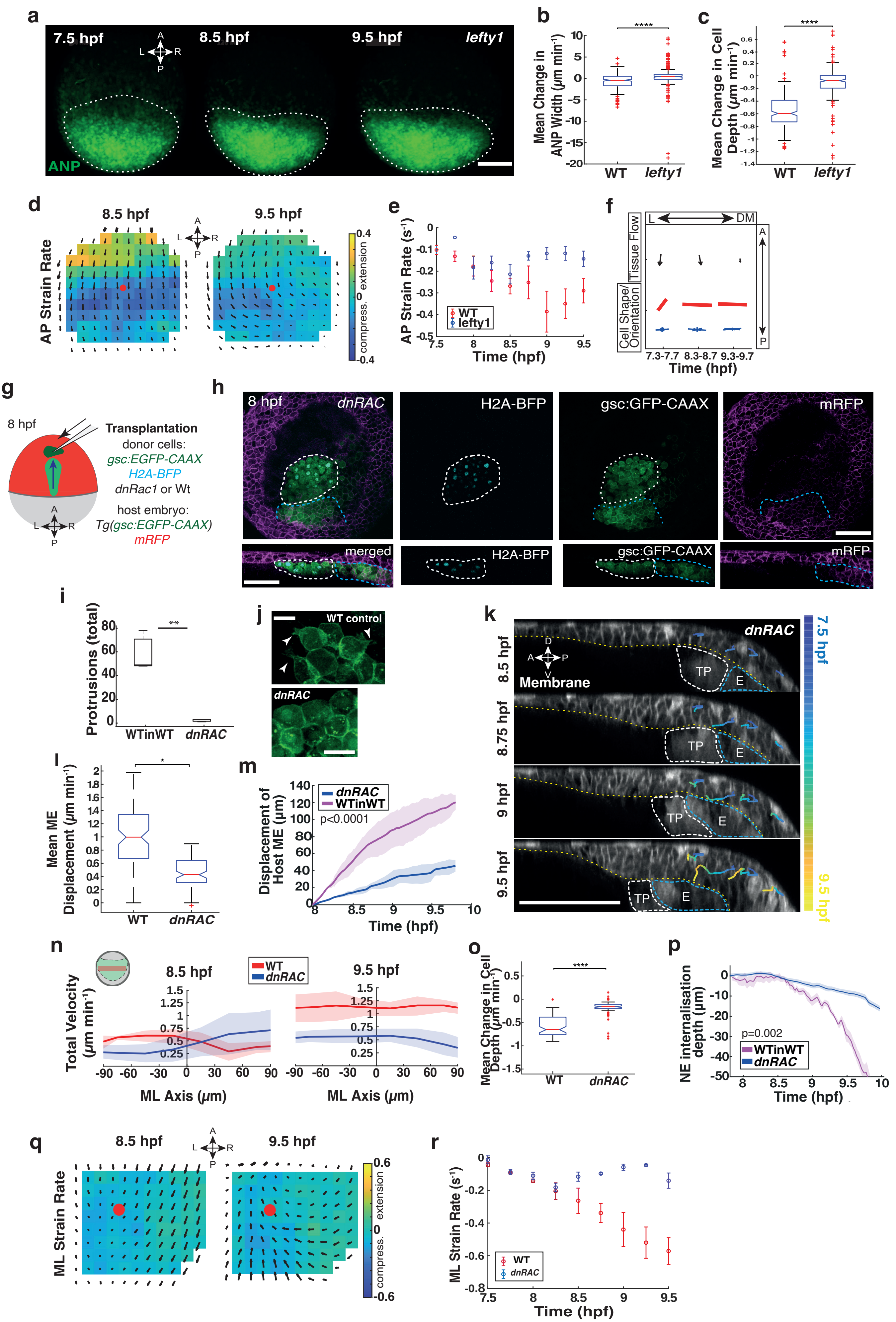

a
7.5 hpf
8.5 hpf
9.5 hpf
lefty1
ANP
b
Mean Change in ANP Width (μm min⁻¹)
WT
lefty1
****
c
Mean Change in Cell Depth (μm min⁻¹)
WT
lefty1
****
d
AP Strain Rate
8.5 hpf
9.5 hpf
extension
compress.
e
AP Strain Rate (s⁻¹)
WT
lefty1
Time (hpf)
f
Tissue Flow
Cell Shape/ Orientation
L
DM
A
P
7.3-7.7
8.3-8.7
9.3-9.7
Time (hpf)
g
8 hpf
Transplantation
donor cells:
gsc:EGFP-CAAX
H2A-BFP
dnRac1 or Wt
host embryo:
Tg(gsc:EGFP-CAAX)
mRFP
h
8 hpf
dnRAC
H2A-BFP
gsc:GFP-CAAX
mRFP
merged
i
Protrusions (total)
WTinWT
dnRAC
**
j
WT control
dnRAC
k
dnRAC
8.5 hpf
8.75 hpf
9 hpf
9.5 hpf
Membrane
TP
E
7.5 hpf
9.5 hpf
l
Mean ME Displacement (μm min⁻¹)
WT
dnRAC
*
m
Displacement of Host ME (μm)
dnRAC
WTinWT
p<0.0001
Time (hpf)
n
Total Velocity (μm min⁻¹)
8.5 hpf
9.5 hpf
WT
dnRAC
ML Axis (μm)
ML Axis (μm)
o
Mean Change in Cell Depth (μm min⁻¹)
WT
dnRAC
****
p
NE internalisation depth (μm)
p=0.002
WTinWT
dnRAC
Time (hpf)
q
ML Strain Rate
8.5 hpf
9.5 hpf
extension
compress.
r
ML Strain Rate (s⁻¹)
WT
dnRAC
Time (hpf)

Supplementary Figure 5. Mesendoderm movement is necessary for neuroectoderm internalisation.

a) Confocal images of neuroectoderm (NE) cells (maximum projections) in the anterior neural plate (ANP) of *Tg(otx2:Venus) lefty1* morphants during gastrulation. White dashes: ANP. **b)** Mean change in ANP width in WT and *lefty 1* morphants. One-way Kruskal-Wallis test ($p=3.3e^{-12}$). **c)** Mean change in NE depth in WT and *lefty 1* morphants (30 cells, 3 embryos). One-way Kruskal-Wallis test ($p=1.7e^{-56}$). **d)** Average ANP domain strains along the anterior-posterior (AP) axis of *lefty1* embryos. Minimum, green (0); maximum stretch, yellow; maximum compression, dark blue. Arrow indicates tissue flows. Red dot: AP axis/ANP leading edge intersection. **e)** Maximum absolute strain rates along the AP axis of WT and *lefty1* morphants during gastrulation. **f)** Tissue flow (top) vs. cell orientation and shape (bottom) from the left half (L) of *lefty1* morphant embryos towards the dorsal midline (DM). Arrows indicate average tissue flow direction and magnitude over 20 minutes either side of the indicated time. Red lines: average cell shape/orientation. Blue lines: histograms of individual cell orientations (15 cells, 3 embryos). **g)** Transplantation schematic. *DnRac1* (dnRAC) expressing ME cells (dark green) transplanted anterior of ME cells (light green) in a host WT embryo before internalisation at 8 hpf. **h)** Confocal images of single and merged channels (xy and zy), showing host *Tg(gsc:EGFP-CAAX)* embryo expressing membrane (mRFP) marker (blue dashes) and transplanted gsc:GFP-CAAX expressing ME cells (white dashes) labelled with a nuclei marker (H2A-BFP). **i)** Total number of protrusions in WT control or *dnRac1* expressing transplanted ME cells. Two-sided t-test (p=0.005). **j)** Multiphoton images of transplanted *dnRac1* expressing or WT control ME cells (gsc-GFP-CAAX). Arrows indicate protrusions in control cells.

k) Sagittal confocal images showing NE cell tracks during gastrulation in host embryos transplanted with *dnRac1* ME cells. Dorsal (D), ventral (V), anterior (A), posterior (P) indicated. Cell membranes (mRFP, grey LUT) labelled. Yellow dashes: NE/ME border. Blue dashes: host ME. White dashes: transplanted ME cells. Tracks coloured blue (7.5 hpf) to yellow (9.5 hpf). **l)** Mean ME leading edge displacement per minute in WT and *dnRAC1* embryos. One-way Kruskal-Wallis test ($p=0.024$). **m)** Anterior displacement of endogenous ME in embryos transplanted with WT control cells (magenta) and with DN Rac1 expressing cells (blue) (n=2 embryos, 5 cells each). One-way Kruskal-Wallis test ($p<0.0001$). **n)** Total velocities at 8.5 hpf and 9.5 hpf along the mediolateral (ML) axis of *dnRAC1* embryos. X-axis: 0 = dorsal midline; negative left, positive right. **o)** Mean change in NE cell depth per minute in WT and *dnRAC1* embryos (30 cells, 3 embryos). One-way Kruskal-Wallis test ($p=2.11e^{-31}$). **p)** NE internalisation depth of dorsal NE layer in embryos transplanted with WT control cells (magenta) and with DN Rac1 expressing cells (blue). One-way Kruskal-Wallis test ($p=0.002$). **q)** Average ANP domain strains along the medio-lateral (ML) axis of *dnRAC1* embryos. Minimum, green (0); maximum stretching, yellow; maximum compression, dark blue. Black arrows: average tissue flows. Red dot: AP axis/ANP leading edge intersection. **r)** Maximum absolute strain rates along the ANP midline along the ML axis of WT and *dnRAC1 emb*ryos during gastrulation. Amount of stretch/compression within each sector is plotted along the y axis. All data are presented as the mean ± SEM of 3 embryos, unless otherwise stated. For box plots, central line indicates the median, and the bottom and top edges of the box indicate the 25th and 75th percentiles, respectively. The whiskers extend to the most extreme data points not considered outliers, and the

outliers are plotted individually using the + marker symbol. Scale bars: 100 μm (a, h, k), 20 μm (j).

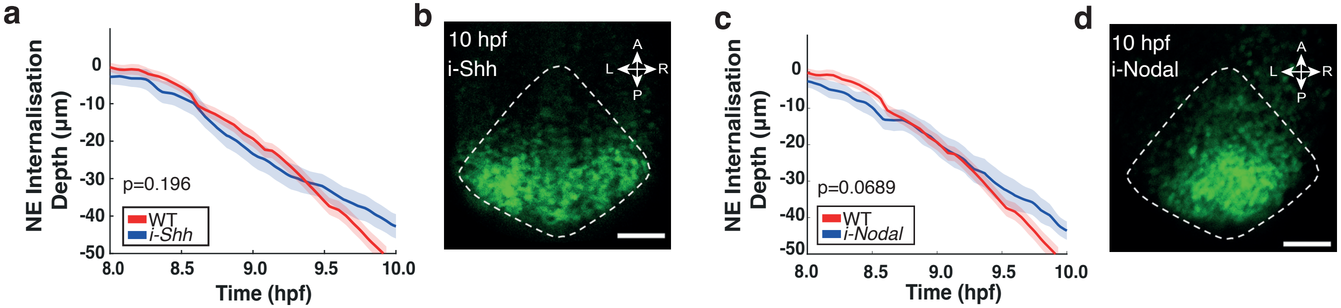
a
NE Internalisation Depth (µm)
0
-10
-20
-30
-40
-50
8.0
8.5
9.0
9.5
10.0
Time (hpf)
p=0.196
WT
i-Shh
b
10 hpf
i-Shh
A
L
R
P
c
NE Internalisation Depth (µm)
0
-10
-20
-30
-40
-50
8.0
8.5
9.0
9.5
10.0
Time (hpf)
p=0.0689
WT
i-Nodal
d
10 hpf
i-Nodal
A
L
R
P

Supplementary Figure 6. Sonic hedgehog (Shh) and Nodal signalling are negligible for neuroectoderm internalisation

a) Neuroectoderm (NE) internalisation depth in WT (red) and Shh inhibited (blue) embryos during gastrulation**.** One-way Kruskal-Wallis test ($p = 0.196$). **b)** Representative confocal image showing ANP shape of Shh inhibited *Tg(otx2:Venus)* embryos at the end of gastrulation (10 hpf). White dashes outline the ANP. Anterior (A), posterior (P), left (L), and right (R) indicated. **c)** NE internalisation depth in WT (red) and Nodal inhibited (blue) embryos during gastrulation. One-way Kruskal-Wallis test ($p = 0.0689$). **d)** Representative confocal image showing ANP shape of Nodal inhibited *Tg(otx2:Venus)* embryos at the end of gastrulation (10 hpf). White dashes outline the ANP. Orientation as in (b). All data are presented as the mean ± SEM of 3 embryos, unless otherwise stated. Scale bars: 100 μm.

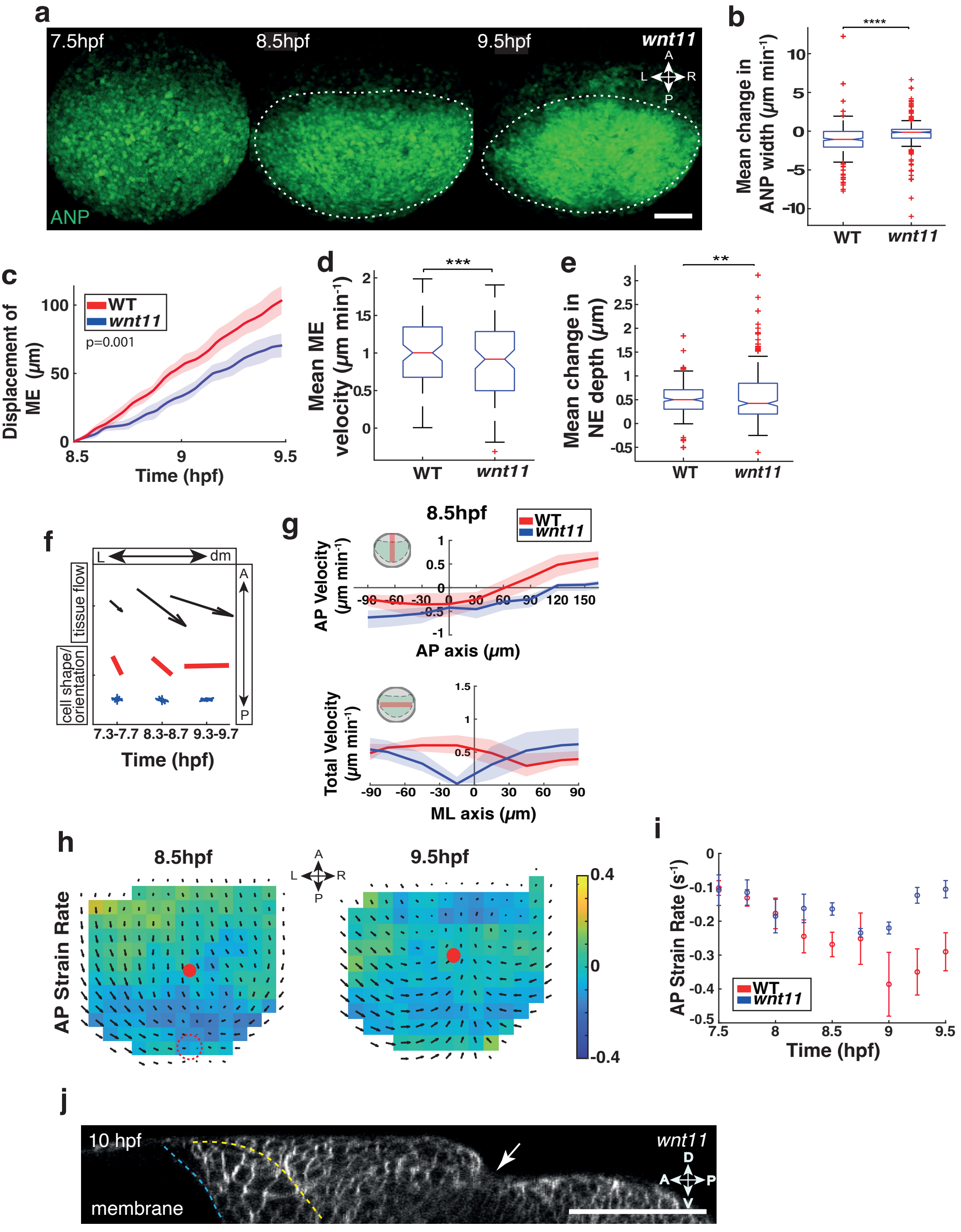

a
7.5hpf
8.5hpf
9.5hpf
wnt11
A
L
R
P
ANP
b
Mean change in ANP width (μm min-1)
WT
wnt11
c
Displacement of ME (μm)
WT
wnt11
p=0.001
Time (hpf)
d
Mean ME velocity (μm min-1)
WT
wnt11
e
Mean change in NE depth (μm)
WT
wnt11
f
L
dm
A
P
tissue flow
cell shape/ orientation
7.3-7.7
8.3-8.7
9.3-9.7
Time (hpf)
g
8.5hpf
WT
wnt11
AP Velocity (μm min-1)
AP axis (μm)
Total Velocity (μm min-1)
ML axis (μm)
h
8.5hpf
9.5hpf
AP Strain Rate
A
L
R
P
i
AP Strain Rate (s-1)
WT
wnt11
Time (hpf)
j
10 hpf
wnt11
membrane
D
A
P
V

Supplementary Figure 7: Convergent extension is required for anterior neural plate tissue shape by driving ventral tissue extension.

a) Confocal images of neuroectoderm (NE) cells (maximum projections) in the ANP of *Tg(otx2:Venus); wnt11* mutant embryos during gastrulation. White dashes: ANP. **b)** Mean change in ANP width in WT and *wnt11* mutants. One-way Kruskal-Wallis test ($p=3.5e^{-9}$). **c)** Anterior displacement of mesendoderm (ME) cells in WT and *wnt11* mutants (30 cells from 3 embryos). One-way Kruskal-Wallis test (p=0.001). **d)** Mean ME leading edge displacement per minute in WT and *wnt11* mutants. One-way Kruskal-Wallis test (p=0.001). **e)** Mean change in NE cell depth per minute in WT and *wnt11* mutants (30 cells, 3 embryos). One-way Kruskal-Wallis test (p=0.009). **f)** Tissue flow direction and magnitude vs cell shape and orientation in the left half (L) of *wnt11* mutant embryos. Arrows indicate average tissue flow direction over 20 minutes either side of the indicated time point. Red line: average cell shape/orientation. Blue line: histograms of individual cell orientations. Anterior (A), posterior (P), left side (L), and dorsal midline (dm) indicated (15 cells, 3 embryos). **g)** Cell velocities at 8.5 hpf in AP direction along dm and total velocities along the mediolateral (ML) axis in WT and *wnt11* mutants. AP velocity, X-axis: 0 marks anterior edge of the ANP; negative, positioned anterior and positive, positioned posterior. Y-axis: negative, posterior and positive, anterior-directed flows. Total velocity, X-axis: 0 marks the dorsal midline; negative, left and positive, right. **h)** Time-averaged ANP domain strain rates along the anterior-posterior (AP) axis of *wnt11* embryos. Minimum, green (0); maximum stretching, yellow; maximum compression, dark blue. Black arrows: time-averaged tissue flows. Red dot marks AP axis/ANP leading edge intersection. Red dashes depict internalisation site. **i)** Maximum absolute strain rates along the and AP axis of

WT and *wnt11* mutants during gastrulation. Negative values indicate compression. **j)** Sagittal confocal image indicating a dorsal kink in the ANP of *wnt11* morphant at 10 hpf. Dorsal up, ventral down, anterior left, posterior right. Cell membranes (mRFP, white) labelled. Yellow dashes: NE/ME interface. Blue dashes: ME/yolk interface. All data are presented as the mean ± SEM of 3 embryos, unless otherwise stated. For box plots, central line indicates the median, and the bottom and top edges of the box indicate the 25th and 75th percentiles, respectively. The whiskers extend to the most extreme data points not considered outliers, and the outliers are plotted individually using the + marker symbol. Scale bars: 100 μm.

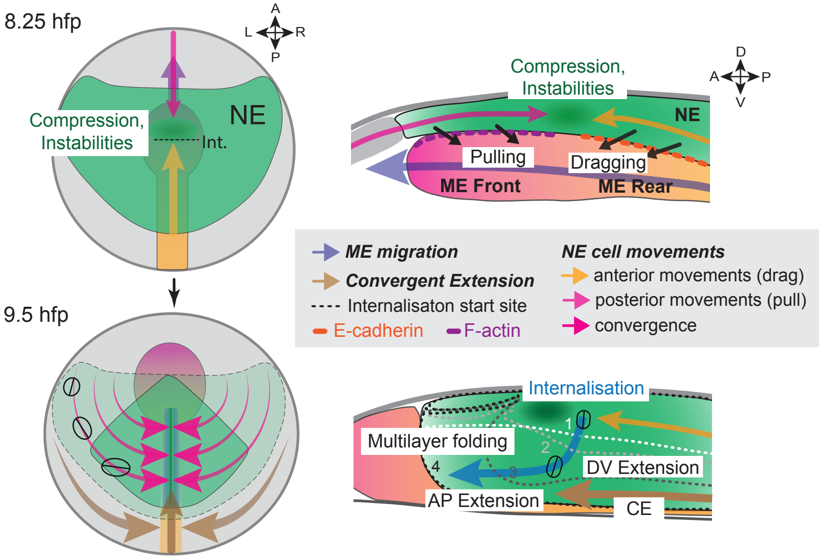

8.25 hfp
A
L
R
P
Compression,
Instabilities
NE
Int.
9.5 hfp
Compression,
Instabilities
D
A
P
V
NE
Pulling
Dragging
ME Front
ME Rear
ME migration
Convergent Extension
Internalisaton start site
E-cadherin
F-actin
NE cell movements
anterior movements (drag)
posterior movements (pull)
convergence
Internalisation
1
2
3
4
Multilayer folding
DV Extension
AP Extension
CE

Supplementary Figure 8: Proposed model of ANP shaping during gastrulation.

Schematic shows a summary of a proposed working model illustrating the mechanisms contributing to ANP tissue morphogenesis during gastrulation. NE, neuroectoderm; ME, mesendoderm.

Supplemental Note for "A multi-tiered mechanical mechanism shapes the early neural plate"

Angus Inman,[1] Elisabeth Spiritosanto,[1] Bridget L. Evans,[1] Judith E. Lutton,[2]
Masazumi Tada,[3] Till Bretschneider,[2] Pierre A. Haas,[4,5,6] and Michael Smutny[1]

[1] *Centre for Mechanochemical Cell Biology and Division of Biomedical Sciences, Warwick Medical School, University of Warwick, Coventry CV4 7AL, United Kingdom*
[2] *Department of Computer Science, University of Warwick, Coventry CV4 7AL, United Kingdom*
[3] *Department of Cell and Developmental Biology, University College London, Gower Street, London WC1E 6BT, United Kingdom*
[4] *Max Planck Institute for the Physics of Complex Systems, Nöthnitzer Straße 38, 01187 Dresden, Germany*
[5] *Max Planck Institute of Molecular Cell Biology and Genetics, Pfotenhauerstraße 108, 01307 Dresden, Germany*
[6] *Center for Systems Biology Dresden, Pfotenhauerstraße 108, 01307 Dresden, Germany*

This Supplemental Note is divided, like Gaul [1], into three parts. In the first part, we give the expressions for the flow singularities used to discuss the experimental flow fields qualitatively. In the second part, we derive the expressions for the regularised singularities used to fit the experimental flow fields and give details of the fitting procedure. In the final part, we discuss modelling tissue compressibility and the YSL/EVL margin and extend the discussion of the main text.

I. FUNDAMENTAL SOLUTIONS OF STOKES FLOW IN TWO DIMENSIONS

In the main text, we analyse the flows in the neuroectoderm qualitatively by discussing the flows of a two-dimensional unbounded incompressible viscous fluid resulting from different point forces (representing migration and drag forces exerted by the mesendoderm) and point sinks (representing ingression or the compressibility of the tissue). Since the Stokes equations that describe these flows are linear, they are the superposition of the flows resulting from the individual point forces and point sources [2]. This Section gives the expressions for these singularities or fundamental solutions.

A. The Stokeslet in two dimensions

We consider the incompressible Stokes equations [2] with a point force $\boldsymbol{f}$ at $\boldsymbol{x} = \boldsymbol{x_0}$. Without loss of generality, we may take $\boldsymbol{x_0} = \boldsymbol{0}$ by translational invariance. The associated flow field is the solution of [2]

$$\boldsymbol{0} = -\boldsymbol{\nabla} p + \nabla^2 \boldsymbol{u} + \boldsymbol{f}\delta(\boldsymbol{x}), \qquad \boldsymbol{\nabla}\cdot\boldsymbol{u} = 0, \tag{1}$$

in which $\boldsymbol{u}(\boldsymbol{x})$ is velocity, $p(\boldsymbol{x})$ is pressure, and where δ denotes the Dirac delta function. Here, and throughout this Supplemental Note, we have nondimensionalised the equations in such a way as to set the fluid viscosity equal to $\mu = 1$.

The solution of Eqs. (1) is called the Stokeslet, and, in two spatial dimensions, it is [2]

$$\boldsymbol{u} = \frac{\boldsymbol{f}}{4\pi}\cdot\left(-\log r\,\boldsymbol{I} + \frac{\boldsymbol{xx}}{r^2}\right), \tag{2}$$

where $r = \|\boldsymbol{x}\|$ and $\boldsymbol{I}$ is the identity. This flow field diverges at $r = 0$ due to the singular nature of the force applied there. Moreover, $\boldsymbol{u}$ diverges unphysically as $r \longrightarrow \infty$; this is known as Stokes' paradox, and is a limitation of the two-dimensional Stokes equations in an unbounded fluid [3]. Owing to this divergence, the Stokeslet has stagnation points on the flow axis defined by $\boldsymbol{f}$. These are clearly unphysical [4], so can be ignored in the qualitative analysis in the main text.

B. Sink flow in two dimensions

The flow resulting from a sink of strength q at $\boldsymbol{x} = \boldsymbol{0}$ in incompressible Stokes flow is described by the equations [2]

$$\boldsymbol{0} = -\boldsymbol{\nabla} p + \nabla^2 \boldsymbol{u}, \qquad \boldsymbol{\nabla}\cdot\boldsymbol{u} = -q\delta(\boldsymbol{x}). \tag{3}$$

The solution of these equations in two dimensions is well-known; it is [2]

$$\boldsymbol{u} = -\frac{q\boldsymbol{x}}{2\pi r^2}. \tag{4}$$

II. FITS OF THE EXPERIMENTAL FLOW FIELDS USING REGULARISED SINGULARITIES

For a more quantitative representation of the experimental flows, we must regularise the divergence of the fundamental solutions at the origin, i.e. at the point where the force is applied or the sink is located, and the divergence in the far-field associated with Stokes' paradox.

To regularise Stokes' paradox, we include a friction force representing the friction between the neuroectoderm and the EVL. To regularise the divergence at the origin, we smear out the point forces and point sinks. The equations (1) and (3) defining the fundamental solutions are thus replaced with

$$\boldsymbol{0} = -\boldsymbol{\nabla} p + \nabla^2 \boldsymbol{u} - \gamma^2\boldsymbol{u} + \boldsymbol{f}\phi_\varepsilon(\boldsymbol{x}), \qquad \boldsymbol{\nabla}\cdot\boldsymbol{u} = 0 \tag{5}$$

and

$$\boldsymbol{0} = -\boldsymbol{\nabla} p + \nabla^2 \boldsymbol{u} - \gamma^2\boldsymbol{u}, \qquad \boldsymbol{\nabla}\cdot\boldsymbol{u} = -q\phi_\varepsilon(\boldsymbol{x}), \tag{6}$$

respectively. In these equations, γ is a friction coefficient, and ϕ_ε is a "blob function", depending on a parameter ε that represents the characteristic distance over which the point force or point sink is smeared out.

The Stokes equations with a friction term $-\gamma^2\boldsymbol{u}$ added in this way are known as the Brinkman equations and also constitute an extension of Darcy's law for flow in porous media [5].

Smearing out force singularities using a blob function ϕ_ε, as in Eqs. (5) and (6), is an efficient technique [6–8] for numerical solution of hydrodynamic equations by regularisation of the singular boundary integral formulation [9]. In these numerical approaches, the blob function is normalised in such a way that

$$\iint_{\mathbb{R}^2} \phi_\varepsilon(\boldsymbol{x})\,\mathrm{d}^2\boldsymbol{x} = 1, \tag{7}$$

and satisfies $\phi_\varepsilon \to \delta$ as $\varepsilon \to 0$, but remain finite for all $\varepsilon > 0$, so that the numerical solution for $\varepsilon > 0$ approaches the exact solution as $\varepsilon \to 0$. In particular, finite values of ε do not therefore have a physical meaning, though they can affect, via the choice of blob function, the rate of convergence of the numerical solution to the exact solution [10].

Here, by contrast, we do ascribe a physical meaning to finite values of ε: they represent the extent over which the mesendoderm exerts friction on the neuroectoderm or over which cells in the neuroectoderm ingress. We are not aware of a prior use of regularised singularities in this way.

In this context, a two-dimensional "Brinkmanlet" solution of Eqs. (5) was derived in Ref. [11] for numerical solution of these equations, but the blob function chosen in Ref. [11] depends on the friction parameter γ. While this is not an issue for these numerical approaches, it is not appropriate in our case, given the physical meaning of the blob function.

Here, we therefore choose the blob function to be the radially symmetric function

$$\phi_\varepsilon(r) = \frac{r\mathcal{K}_1(r/\varepsilon)}{4\pi\varepsilon^3}, \tag{8a}$$

where we use $\mathcal{K}_0, \mathcal{K}_1, \dots$ to denote the modified Bessel functions of the second kind [12]. It satisfies the normalisation condition (7). With the hindsight of the calculations that follow below, this somewhat arcane choice of blob function is motivated by the simple form of its Fourier transform [13]

$$\tilde{\phi}_\varepsilon(k) = \left(1 + \varepsilon^2 k^2\right)^{-2}, \tag{8b}$$

where we have let $k = \|\boldsymbol{k}\|$. This allows the Fourier inversion integrals that arise in the solution of Eqs. (5) and (6) to be computed in closed form.

A. Solution of Eqs. (5): the regularised "Brinkmanlet"

We solve Eqs. (5) in Fourier space. Taking their Fourier transform [13], we find

$$\boldsymbol{0} = -\mathrm{i}\boldsymbol{k}\tilde{p} - k^2\tilde{\boldsymbol{u}} - \gamma^2\tilde{\boldsymbol{u}} + \boldsymbol{f}\tilde{\phi}_\varepsilon(k), \qquad \mathrm{i}\boldsymbol{k}\cdot\tilde{\boldsymbol{u}} = 0, \tag{9}$$

for a radially symmetric blob function, as in Eq. (8a). Dotting the first equation with $\boldsymbol{k}$ and using the second one yields

$$\tilde{p} = \boldsymbol{f}\cdot\left(-\frac{\mathrm{i}\boldsymbol{k}\tilde{\phi}_\varepsilon(k)}{k^2}\right), \tag{10a}$$

and hence, on substituting this into the first of Eqs. (9),

$$\tilde{\boldsymbol{u}} = \boldsymbol{f}\cdot\left[\frac{\tilde{\phi}_\varepsilon(k)}{k^2+\gamma^2}\left(\boldsymbol{I} - \frac{\boldsymbol{kk}}{k^2}\right)\right]. \tag{10b}$$

By symmetry, the regularised "Brinkmanlet" solution is thus

$$\boldsymbol{u} = \boldsymbol{f}\cdot\left[A_\varepsilon(r)\boldsymbol{I} + B_\varepsilon(r)\frac{\boldsymbol{xx}}{r^2}\right], \tag{11}$$

in which the coefficients $A_\varepsilon(r), B_\varepsilon(r)$ remain to be determined by computing the Fourier inversion

$$A_\varepsilon(r)\boldsymbol{I} + B_\varepsilon(r)\frac{\boldsymbol{xx}}{r^2} = \frac{1}{(2\pi)^2}\iint_{\mathbb{R}^2}\frac{\tilde{\phi}_\varepsilon(k)}{k^2+\gamma^2}\left(\boldsymbol{I} - \frac{\boldsymbol{kk}}{k^2}\right)\mathrm{e}^{\mathrm{i}\boldsymbol{k}\cdot\boldsymbol{x}}\,\mathrm{d}^2\boldsymbol{k}. \tag{12}$$

With Cartesian axes in which $\boldsymbol{x} = (r, 0)$, $\boldsymbol{k} = k(\cos\theta, \sin\theta)$, taking the trace and the $(1,1)$ component of Eq. (12) respectively, and using results from the Appendix of this Note,

$$2A_\varepsilon(r) + B_\varepsilon(r) = \frac{1}{(2\pi)^2}\int_0^\infty\!\!\int_0^{2\pi}\frac{\tilde{\phi}_\varepsilon(k)}{k^2+\gamma^2}\mathrm{e}^{\mathrm{i}kr\cos\theta}\,k\,\mathrm{d}\phi\,\mathrm{d}k = \frac{1}{2\pi}\int_0^\infty\frac{k\tilde{\phi}_\varepsilon(k)}{k^2+\gamma^2}\mathcal{J}_0(kr)\,\mathrm{d}k, \tag{13a}$$

$$A_\varepsilon(r) + B_\varepsilon(r) = \frac{1}{(2\pi)^2}\int_0^\infty\!\!\int_0^{2\pi}\frac{\tilde{\phi}_\varepsilon(k)}{k^2+\gamma^2}\sin^2\theta\,\mathrm{e}^{\mathrm{i}kr\cos\theta}\,k\,\mathrm{d}\phi\,\mathrm{d}k = \frac{1}{2\pi r}\int_0^\infty\frac{\tilde{\phi}_\varepsilon(k)}{k^2+\gamma^2}\mathcal{J}_1(kr)\,\mathrm{d}k, \tag{13b}$$

where we use $\mathcal{J}_0, \mathcal{J}_1, \dots$ to denote the Bessel functions of the first kind [12]. We now specialise to the blob function (8a). Noting the partial fraction decomposition [14]

$$\frac{\tilde{\phi}_\varepsilon(k)}{k^2+\gamma^2} = \frac{1}{\left(\gamma^2\varepsilon^2-1\right)^2\left(k^2+\gamma^2\right)} - \frac{\varepsilon^2}{\left(\gamma^2\varepsilon^2-1\right)^2\left(\varepsilon^2k^2+1\right)} + \frac{\varepsilon^2}{\left(\gamma^2\varepsilon^2-1\right)\left(\varepsilon^2k^2+1\right)^2}, \tag{14}$$

and using more integral identities from the Appendix, we find

$$2A_\varepsilon(r) + B_\varepsilon(r) = \frac{1}{4\pi\left(\gamma^2\varepsilon^2-1\right)}\left[\frac{2\mathcal{K}_0(\gamma r) - 2\mathcal{K}_0(r/\varepsilon)}{\gamma^2\varepsilon^2-1} + \frac{r}{\varepsilon}\mathcal{K}_1(r/\varepsilon)\right], \tag{15a}$$

$$A_\varepsilon(r) + B_\varepsilon(r) = \frac{1}{2\pi r^2\gamma^2} - \frac{1}{4\pi\left(\gamma^2\varepsilon^2-1\right)}\left[\frac{2}{r\left(\gamma^2\varepsilon^2-1\right)}\left(\frac{\mathcal{K}_1(\gamma r)}{\gamma} - \varepsilon\mathcal{K}_1(r/\varepsilon)\right) + \mathcal{K}_2(r/\varepsilon)\right]. \tag{15b}$$

This is a system of simultaneous equations for $A_\varepsilon(r), B_\varepsilon(r)$, with solution

$$A_\varepsilon(r) = -\frac{1}{2\pi r^2\gamma^2} + \frac{1}{4\pi\left(\gamma^2\varepsilon^2-1\right)^2}\left[2\mathcal{K}_0(\gamma r) + \frac{2\mathcal{K}_1(\gamma r)}{\gamma r} + (\gamma^2\varepsilon^2-3)\mathcal{K}_0(r/\varepsilon) + \frac{2\gamma^2\varepsilon^4 + \left(\gamma^2r^2-4\right)\varepsilon^2 - r^2}{\varepsilon r}\mathcal{K}_1(r/\varepsilon)\right], \tag{16a}$$

$$B_\varepsilon(r) = \frac{1}{\pi r^2\gamma^2} - \frac{1}{4\pi\left(\gamma^2\varepsilon^2-1\right)^2}\left[2(\gamma^2\varepsilon^2-2)\mathcal{K}_0(r/\varepsilon) + \frac{4\gamma^2\varepsilon^4+\left(\gamma^2r^2-8\right)\varepsilon^2-r^2}{\varepsilon r}\mathcal{K}_1(r/\varepsilon)+2\mathcal{K}_2(\gamma r)\right], \tag{16b}$$

where we have used $\mathcal{K}_2(x) = \mathcal{K}_0(x) + 2\mathcal{K}_1(x)/x$ [12]. The singular limit $\varepsilon\gamma = 1$ is regular: using Ref. [12], we find

$$A_{\gamma^{-1}}(r) = -\frac{1}{2\pi r^2\gamma^2} + \frac{1}{16\pi}\left[\left(\gamma^2r^2+4\right)\mathcal{K}_0(\gamma r) + \left(3\gamma^2r^2+8\right)\frac{\mathcal{K}_1(\gamma r)}{\gamma r}\right], \tag{17a}$$

$$B_{\gamma^{-1}}(r) = \frac{1}{\pi r^2\gamma^2} - \frac{1}{16\pi}\left[\left(\gamma^2r^2+8\right)\mathcal{K}_0(\gamma r) + 4\left(\gamma^2r^2+4\right)\frac{\mathcal{K}_1(\gamma r)}{\gamma r}\right]. \tag{17b}$$

This determines the Brinkmanlet (11) completely. The exponential decay of $\mathcal{K}_0, \mathcal{K}_1, \dots$ for $r \to \infty$ [12] implies

$$\boldsymbol{u} \sim \frac{1}{2\pi r^2\gamma^2}\left(-\boldsymbol{f} + \frac{2(\boldsymbol{f}\cdot\boldsymbol{x})\boldsymbol{x}}{r^2}\right). \tag{18}$$

In particular, $\boldsymbol{u} \to \boldsymbol{0}$ as $r \to \infty$: There is no Stokes' paradox. Equation (18) also shows that, in the far field, $\boldsymbol{u} \parallel -\boldsymbol{f}$ in the direction $\boldsymbol{f}\cdot\boldsymbol{x} = 0$. Clearly, $\boldsymbol{u} \parallel \boldsymbol{f}$ close to the origin (where the force is aplied the fluid), so this is a signature of lateral vortices in the flow field.

B. Solution of Eqs. (6): the regularised Brinkman sink

We solve Eqs. (6), again, in Fourier space. Taking their Fourier transform [13], we find, for a radially symmetric blob function,

$$\boldsymbol{0} = -\mathrm{i}\boldsymbol{k}\tilde{p} - k^2\tilde{\boldsymbol{u}} - \gamma^2\tilde{\boldsymbol{u}}, \qquad \mathrm{i}\boldsymbol{k}\cdot\tilde{\boldsymbol{u}} = -q\tilde{\phi}_\varepsilon(k). \tag{19}$$

Contracting the first equation with $\boldsymbol{k}$, using the second equation, and backsubstituting into the first now yields

$$\tilde{p} = -\left(1+\frac{\gamma^2}{k^2}\right)q\tilde{\phi}_\varepsilon(k) \quad\text{and}\quad \tilde{\boldsymbol{u}} = \frac{\mathrm{i}\boldsymbol{k}}{k^2}q\tilde{\phi}_\varepsilon(k). \tag{20}$$

By symmetry, the regularised Brinkman sink flow is

$$\boldsymbol{u} = C_\varepsilon(r)\boldsymbol{x} = \frac{1}{(2\pi)^2}\iint_{\mathbb{R}^2}\left(\frac{\mathrm{i}\boldsymbol{k}}{k^2}q\tilde{\phi}_\varepsilon(k)\right)\mathrm{e}^{\mathrm{i}\boldsymbol{k}\cdot\boldsymbol{x}}\,\mathrm{d}^2\boldsymbol{k}. \tag{21}$$

With Cartesian axes in which $\boldsymbol{x} = (r, 0)$, $\boldsymbol{k} = k(\cos\theta, \sin\theta)$, the non-zero component of this relation yields, using the blob function in Eq. (8a),

$$\begin{aligned} C_\varepsilon(r) &= \frac{1}{(2\pi)^2 r}\int_0^\infty\int_0^{2\pi}\left(\mathrm{i}q\tilde{\phi}_\varepsilon(k)\cos\theta\right)\mathrm{e}^{\mathrm{i}kr\cos\theta}\,\mathrm{d}\theta\,\mathrm{d}k \\ &= \frac{-q}{2\pi r}\int_0^\infty\frac{\mathcal{J}_1(kr)}{\left(\varepsilon^2k^2+1\right)^2}\,\mathrm{d}k = \frac{-q}{2\pi r^2}\left[1-\frac{r^2}{2\varepsilon^2}\mathcal{K}_2(r/\varepsilon)\right], \end{aligned} \tag{22}$$

where we have used integral identities from the Appendix. This determines the solution (21) of Eqs. (6) completely. We emphasise that the flow field is independent of the friction coefficient γ, though the pressure field depends on γ.

C. Fitting experimental flow fields

To fit the experimental flow field $\boldsymbol{U}(X, Y)$, where the X, Y directions are left $\to$ right and vegetal $\to$ animal, respectively, we first determine the axis of mesendoderm migration $X = X_0$. We do so by minimising $\|\boldsymbol{U}(X, Y) - \boldsymbol{U}(2X_0 - X, Y)\|$ with respect to X_0 using the MATLAB (The MathWorks, Inc.) function `fminsearch`, linearly interpolating the experimental flow fields onto a regular grid.

We then shift the coordinates so that $X_0 = 0$, and again interpolate $\boldsymbol{U}(X, Y)$ linearly on a regular grid (X_i, Y_j) for $i, j = 1, 2, \dots, N = 20$. We define $\boldsymbol{U_{i,j}} = \boldsymbol{U}(X_i, Y_j)$, and we decompose

$$\boldsymbol{U_{i,j}} = \overline{\boldsymbol{U}}_{\boldsymbol{i,j}} + \hat{\boldsymbol{U}}_{\boldsymbol{i,j}}, \quad \text{with } \overline{\boldsymbol{U}}_{\boldsymbol{i,j}} = \frac{\boldsymbol{U_{i,j}} + \boldsymbol{U_{N-i,j}}}{2}, \tag{23}$$

for $i, j = 1, 2, \dots, N$, so that $\overline{\boldsymbol{U}}_{\boldsymbol{i,j}}$ and $\hat{\boldsymbol{U}}_{\boldsymbol{i,j}}$ are, respectively, the left-right symmetric and asymmetric parts of the flow.

To fit the symmetric part of the experimental flow, we represent sinks and forces on and parallel to the axis of mesendoderm migration by a regularised Brinkman sink flow $\boldsymbol{u_0}$ of strength q at $(0, y_0)$ and two regularised Brinkmanlet flows $\boldsymbol{u_1}, \boldsymbol{u_2}$ resulting from forces $\boldsymbol{f_1} = (0, f_1)$, $\boldsymbol{f_2} = (0, f_2)$ at $(0, y_1)$, $(0, y_2)$, respectively. These regularised singularities result in a flow $\boldsymbol{u} = \boldsymbol{u_0} + \boldsymbol{u_1} + \boldsymbol{u_2}$, and we let $\boldsymbol{u_{i,j}} = \boldsymbol{u}(X_i, Y_j)$, for $i, j = 1, 2, \dots, N$. The fitting minimises the relative error

$$E = \sum_{i=1}^N\sum_{j=1}^N\left(\max\left\{\frac{\|\boldsymbol{u_{i,j}} - \overline{\boldsymbol{U}}_{\boldsymbol{i,j}}\| - \|\hat{\boldsymbol{U}}_{\boldsymbol{i,j}}\|}{\|\overline{\boldsymbol{U}}_{\boldsymbol{i,j}}\|}, 0\right\}\right)^2 \tag{24}$$

with respect to $y_0, y_1, y_2, q, f_1, f_2$, the friction parameter γ, and the regularisations $\varepsilon_0, \varepsilon_1 = \varepsilon_2$ of the three singularities using `fminsearch` [15]. Cutting off the relative error in this way ensures that differences between the fitted and observed flows below the asymmetry of the experimental data (as a proxy for experimental error) are not penalised. One could argue that minimising a relative rather than an absolute error puts too much weight on small flow velocities (the measurements of which may be unreliable), but we found that the fits minimising an absolute error tend to not represent topological features (e.g. vortices) of the experimental flows (not shown).

III. EXTENSIONS OF THE MODEL

In this Section, we discuss extensions of the model to represent the YSL/EVL margin and the effects of compressibility.

A. Modelling the YSL/EVL margin

We represent the YSL/EVL margin as a boundary at $Y = Y_\mathrm{m}$, at which we apply the boundary condition $\boldsymbol{u}\cdot\hat{\boldsymbol{Y}} = 0$, allowing flow parallel to, but not across the margin.

We add (regularised) image singularities to the flow: an image Brinkman sink flow $\boldsymbol{u}_0'$ of strength q at $(0, 2Y_\mathrm{m} - y_0)$, and image Brinkmanlet flows $\boldsymbol{u}_1', \boldsymbol{u}_2'$ from forces $(0, -f_1), (0, -f_2)$ at the respective points $(0, 2Y_\mathrm{m} - y_1), (0, 2Y_\mathrm{m} - y_2)$. The resulting total flow $\boldsymbol{u} = \boldsymbol{u}_0 + \boldsymbol{u}_1 + \boldsymbol{u}_2 + \boldsymbol{u}_0' + \boldsymbol{u}_1' + \boldsymbol{u}_2'$ satisfies the boundary condition $\boldsymbol{u} \cdot \hat{\boldsymbol{Y}} = 0$ by symmetry. We emphasise that, given Y_m, modelling the YSL/EVL margin does not add additional parameters to be fitted to the model.

B. Compressibility

A major simplifying assumption of our model is to assume the two-dimensional experimental flow to be incompressible: the cell sheet is essentially incompressible (since cells are mostly water), but in-plane compression of the cell sheet can be balanced by changes in its thickness, so incompressibility of the three-dimensional flow does not imply incompressibility of the two-dimensional flow. We do believe, however, that it is appropriate nevertheless to use an incompressible model since the compressive flows co-localise with the ingression of the cell sheet, so that the sink flow in the model can be interpreted as the sum of a contribution from compressibility of the flow, and the actual ingression flow.

Still, to discuss the effect of compressibility further, we derive here the expressions for (regularised) force singularities in a very compressible Brinkman flow. We start from the steady Cauchy equation without inertia [16], with a friction force $-\gamma^2\boldsymbol{u}$ and a body force $\boldsymbol{F}(\boldsymbol{x})$,

$$\boldsymbol{0} = \boldsymbol{\nabla} \cdot \boldsymbol{\sigma} - \gamma^2 \boldsymbol{u} + \boldsymbol{F}(\boldsymbol{x}), \tag{25a}$$

where $\boldsymbol{\sigma}$ is the stress tensor. For a compressible fluid [17],

$$\boldsymbol{\nabla} \cdot \boldsymbol{\sigma} = -\boldsymbol{\nabla} p + \nabla^2 \boldsymbol{u} + \alpha \boldsymbol{\nabla}(\boldsymbol{\nabla} \cdot \boldsymbol{u}), \tag{25b}$$

wherein α is the ratio of bulk to shear viscosity. The steady continuity equation with a sink $Q(\boldsymbol{x})$ is [18]

$$\boldsymbol{\nabla} \cdot (\rho \boldsymbol{u}) = -Q(\boldsymbol{x}), \tag{25c}$$

in which $\rho(\boldsymbol{x})$ is the density of the fluid, which is coupled to the pressure via an equation of state $p = P(\rho)$ [19]. Importantly, Eq. (25c) is nonlinear, so the solution of problem (25) cannot be expressed as a superposition of singularities.

Progress can however made in the particular case of a very compressible fluid with $p \approx 0$ [19], in which case Eq. (25a) decouples from Eq. (25c) and becomes linear. The assumption $p \approx 0$ was previously made to describe the forces positioning the neural anlage in zebrafish [20]. Here, we will thus seek (regularised) force singularities such that

$$\boldsymbol{0} = \nabla^2 \boldsymbol{u} + \alpha \boldsymbol{\nabla}(\boldsymbol{\nabla} \cdot \boldsymbol{u}) - \gamma^2 \boldsymbol{u} + \boldsymbol{f} \phi_\varepsilon(r). \tag{26}$$

We solve this equation, again, in Fourier space [13]. We find, again assuming the blob function to be radially symmetric,

$$\boldsymbol{0} = -k^2 \tilde{\boldsymbol{u}} - \alpha \boldsymbol{k}(\boldsymbol{k} \cdot \tilde{\boldsymbol{u}}) - \gamma^2 \tilde{\boldsymbol{u}} + \boldsymbol{f} \tilde{\phi}_\varepsilon(k). \tag{27}$$

Contracting this equation with $\boldsymbol{k}$ yields

$$\boldsymbol{k} \cdot \tilde{\boldsymbol{u}} = \frac{\tilde{\phi}_\varepsilon(k)}{(1+\alpha)k^2 + \gamma^2} \boldsymbol{f} \cdot \boldsymbol{k}, \tag{28a}$$

whence

$$\tilde{\boldsymbol{u}} = \boldsymbol{f} \cdot \left[\frac{\tilde{\phi}_\varepsilon(k)}{k^2 + \gamma^2} \left(\boldsymbol{I} - \frac{\alpha \boldsymbol{k}\boldsymbol{k}}{(1+\alpha)k^2 + \gamma^2} \right) \right]. \tag{28b}$$

The regularised compressible Brinkmanlet solution in two dimensions is therefore

$$\boldsymbol{u} = \boldsymbol{f} \cdot \left[D_\varepsilon(r) \boldsymbol{I} + E_\varepsilon(r) \frac{\boldsymbol{x}\boldsymbol{x}}{r^2} \right] \equiv \boldsymbol{f} \cdot \boldsymbol{U}_\varepsilon(\boldsymbol{x}), \tag{29}$$

where the coefficients $D_\varepsilon(r), E_\varepsilon(r)$ are determined by the Fourier inversion integral

$$\boldsymbol{U}_\varepsilon(\boldsymbol{x}) = \frac{1}{(2\pi)^2} \iint_{\mathbb{R}^2} \frac{\tilde{\phi}_\varepsilon(k)}{k^2 + \gamma^2} \left(\boldsymbol{I} - \frac{\alpha \boldsymbol{k}\boldsymbol{k}}{(1+\alpha)k^2 + \gamma^2} \right) \mathrm{e}^{\mathrm{i}\boldsymbol{k}\cdot\boldsymbol{x}} \, \mathrm{d}^2\boldsymbol{k}. \tag{30}$$

Similarly to the derivation of Eqs. (13), it follows that

$$2D_\varepsilon(r) + E_\varepsilon(r) = \frac{1}{2\pi} \int_0^\infty \frac{k \tilde{\phi}_\varepsilon(k)}{k^2 + \gamma^2} \left[2 - \frac{\alpha k^2}{(1+\alpha)k^2 + \gamma^2} \right] \mathcal{J}_0(kr) \, \mathrm{d}k, \tag{31a}$$

$$D_\varepsilon(r) + E_\varepsilon(r) = \frac{1}{2\pi} \int_0^\infty \frac{k \tilde{\phi}_\varepsilon(k)}{k^2 + \gamma^2} \left\{ \left[1 - \frac{\alpha k^2}{(1+\alpha)k^2 + \gamma^2} \right] \mathcal{J}_0(kr) + \frac{\alpha k \mathcal{J}_1(kr)}{r\left[(1+\alpha)k^2 + \gamma^2\right]} \right\} \mathrm{d}k. \tag{31b}$$

For the blob function in Eq. (8a), using the partial fraction decompositions

$$\begin{aligned}\frac{\tilde{\phi}_\varepsilon(k)}{k^2 + \gamma^2} \left[2 - \frac{\alpha k^2}{(1+\alpha)k^2 + \gamma^2} \right] = {} & \frac{(1+\alpha)^2}{\left(\gamma^2\varepsilon^2 - 1 - \alpha\right)^2 \left[(1+\alpha)k^2 + \gamma^2\right]} + \frac{\varepsilon^2\left(2\gamma^2\varepsilon^2 - 2 - \alpha\right)}{\left(\gamma^2\varepsilon^2 - 1\right)\left(\gamma^2\varepsilon^2 - 1 - \alpha\right)\left(\varepsilon^2k^2 + 1\right)^2} \\ & + \frac{1}{\left(\gamma^2\varepsilon^2 - 1\right)^2\left(k^2 + \gamma^2\right)} - \frac{\varepsilon^2\left[(1+\alpha)(2+\alpha) - 4(1+\alpha)\gamma^2\varepsilon^2 + (\alpha+2)\gamma^4\varepsilon^4\right]}{\left(\gamma^2\varepsilon^2 - 1\right)^2\left(\gamma^2\varepsilon^2 - 1 - \alpha\right)^2\left(\varepsilon^2k^2 + 1\right)},\end{aligned} \tag{32a}$$

$$\begin{aligned}\frac{\tilde{\phi}_\varepsilon(k)}{k^2 + \gamma^2} \left[1 - \frac{\alpha k^2}{(1+\alpha)k^2 + \gamma^2} \right] = {} & \frac{(1+\alpha)^2}{\left(\gamma^2\varepsilon^2 - 1 - \alpha\right)^2 \left[(1+\alpha)k^2 + \gamma^2\right]} - \frac{(1+\alpha)\varepsilon^2}{\left(\gamma^2\varepsilon^2 - 1 - \alpha\right)^2\left(\varepsilon^2k^2 + 1\right)} \\ & + \frac{\varepsilon^2}{\left(\gamma^2\varepsilon^2 - 1 - \alpha\right)\left(\varepsilon^2k^2 + 1\right)^2},\end{aligned} \tag{32b}$$

$$\frac{\alpha k^2\tilde{\phi}_\varepsilon(k)}{(k^2+\gamma^2)\left[(1+\alpha)k^2+\gamma^2\right]} = \frac{1}{(\gamma^2\varepsilon^2-1)^2(k^2+\gamma^2)} - \frac{(1+\alpha)^2}{(\gamma^2\varepsilon^2-1-\alpha)^2\left[(1+\alpha)k^2+\gamma^2\right]} - \frac{\alpha\varepsilon^2}{(\gamma^2\varepsilon^2-1)(\gamma^2\varepsilon^2-1-\alpha)(\varepsilon^2k^2+1)^2} + \frac{\alpha\varepsilon^2(\gamma^4\varepsilon^4-1-\alpha)}{(\gamma^2\varepsilon^2-1)^2(\gamma^2\varepsilon^2-1-\alpha)^2(\varepsilon^2k^2+1)}, \tag{32c}$$

and integrals from the Appendix, we obtain

$$2D_\varepsilon(r)+E_\varepsilon(r) = \frac{\mathcal{K}_0(\gamma r)}{2\pi(\gamma^2\varepsilon^2-1)^2} + \frac{(1+\alpha)\mathcal{K}_0\left(\frac{\gamma r}{\sqrt{1+\alpha}}\right)}{2\pi(\gamma^2\varepsilon^2-1-\alpha)^2} - \frac{\left[(1+\alpha)(2+\alpha)-4(1+\alpha)\gamma^2\varepsilon^2+(\alpha+2)\gamma^4\varepsilon^4\right]\mathcal{K}_0(r/\varepsilon)}{2\pi(\gamma^2\varepsilon^2-1)^2(\gamma^2\varepsilon^2-1-\alpha)^2} + \frac{(2\gamma^2\varepsilon^2-2-\alpha)\,r\mathcal{K}_1(r/\varepsilon)}{4\pi\varepsilon(\gamma^2\varepsilon^2-1)(\gamma^2\varepsilon^2-1-\alpha)}, \tag{33a}$$

$$D_\varepsilon(r)+E_\varepsilon(r) = \frac{(1+\alpha)\mathcal{K}_0\left(\frac{\gamma r}{\sqrt{1+\alpha}}\right)}{2\pi(\gamma^2\varepsilon^2-1-\alpha)^2} - \frac{\left[(\alpha+2)\gamma^2\varepsilon^2+(\alpha+1)(\alpha-2)\right]\mathcal{K}_0(r/\varepsilon)}{2\pi(\gamma^2\varepsilon^2-1)(\gamma^2\varepsilon^2-1-\alpha)^2} - \frac{\mathcal{K}_1(\gamma r)}{2\pi\gamma r(\gamma^2\varepsilon^2-1)^2} + \frac{(1+\alpha)^{3/2}\mathcal{K}_1\left(\frac{\gamma r}{\sqrt{1+\alpha}}\right)}{2\pi\gamma r(\gamma^2\varepsilon^2-1-\alpha)^2} + \left\{\frac{\alpha\varepsilon\left[2(1+\alpha)-(2+\alpha)\gamma^2\varepsilon^2\right]}{r(\gamma^2\varepsilon^2-1-\alpha)(\gamma^2\varepsilon^2-1)^2} + \frac{r}{2\varepsilon}\right\}\frac{\mathcal{K}_1(r/\varepsilon)}{2\pi(\gamma^2\varepsilon^2-1-\alpha)}, \tag{33b}$$

where, again, we have used $\mathcal{K}_2(x) = \mathcal{K}_0(x) + 2\mathcal{K}_1(x)/x$ [12]. Thence

$$D_\varepsilon(r) = \frac{\mathcal{K}_0(\gamma r)}{2\pi(\gamma^2\varepsilon^2-1)^2} - \frac{\left[(2+\alpha)\gamma^2\varepsilon^2-2-3\alpha\right]\mathcal{K}_0(r/\varepsilon)}{4\pi(\gamma^2\varepsilon^2-1-\alpha)(\gamma^2\varepsilon^2-1)^2} + \frac{\mathcal{K}_1(\gamma r)}{2\pi\gamma r(\gamma^2\varepsilon^2-1)^2} - \frac{(1+\alpha)^{3/2}\mathcal{K}_1\left(\frac{\gamma r}{\sqrt{1+\alpha}}\right)}{2\pi\gamma r(\gamma^2\varepsilon^2-1-\alpha)^2} + \left\{\frac{\alpha\varepsilon\left[(2+\alpha)\gamma^2\varepsilon^2-2(1+\alpha)\right]}{r(\gamma^2\varepsilon^2-1-\alpha)^2(\gamma^2\varepsilon^2-1)} + \frac{r}{2\varepsilon}\right\}\frac{\mathcal{K}_1(r/\varepsilon)}{2\pi(\gamma^2\varepsilon^2-1)}, \tag{34a}$$

$$E_\varepsilon(r) = \frac{\alpha\left[2(1+\alpha)-(2+\alpha)\gamma^2\varepsilon^2\right]\mathcal{K}_0(r/\varepsilon)}{2\pi(\gamma^2\varepsilon^2-1-\alpha)^2(\gamma^2\varepsilon^2-1)^2} + \left\{\frac{\varepsilon\left[4(1+\alpha)-2(2+\alpha)\gamma^2\varepsilon^2\right]}{r(\gamma^2\varepsilon^2-1-\alpha)(\gamma^2\varepsilon^2-1)} + \frac{r}{2\varepsilon}\right\}\frac{\alpha\mathcal{K}_1(r/\varepsilon)}{2\pi(\gamma^2\varepsilon^2-1-\alpha)(\gamma^2\varepsilon^2-1)} - \frac{\mathcal{K}_2(\gamma r)}{2\pi(\gamma^2\varepsilon^2-1)^2} + \frac{(1+\alpha)\mathcal{K}_2\left(\frac{\gamma r}{\sqrt{1+\alpha}}\right)}{2\pi(\gamma^2\varepsilon^2-1-\alpha)^2}. \tag{34b}$$

The singular limits $\varepsilon\gamma = 1$ and $\varepsilon\gamma = \sqrt{1+\alpha}$ are, again, regular, and we find, using Ref. [12],

$$D_{\gamma^{-1}}(r) = \frac{1}{16\pi\alpha^2}\left\{\left[4\alpha(1+\alpha)+\alpha^2r^2\gamma^2\right]\mathcal{K}_0(\gamma r) + \left[\frac{8(1+\alpha)^2}{\gamma r}+3\alpha^2\gamma r\right]\mathcal{K}_1(\gamma r) - \frac{8(1+\alpha)^{3/2}}{\gamma r}\mathcal{K}_1\left(\frac{\gamma r}{\sqrt{1+\alpha}}\right)\right\}, \tag{35a}$$

$$E_{\gamma^{-1}}(r) = \frac{1}{16\pi\alpha^2}\left\{-\left[8(1+\alpha)^2+\alpha^2r^2\gamma^2\right]\mathcal{K}_0(\gamma r) - 4(1+\alpha)\left[4(1+\alpha)+\alpha\gamma^2r^2\right]\frac{\mathcal{K}_1(\gamma r)}{\gamma r} + 8(1+\alpha)\mathcal{K}_2\left(\frac{\gamma r}{\sqrt{1+\alpha}}\right)\right\}, \tag{35b}$$

$$D_{\frac{\sqrt{1+\alpha}}{\gamma}}(r) = \frac{1}{16\pi\alpha^2}\left[8\mathcal{K}_0(\gamma r) + \frac{8\mathcal{K}_1(\gamma r)}{\gamma r} - \frac{4(2+\alpha)}{1+\alpha}\mathcal{K}_0\left(\frac{\gamma r}{\sqrt{1+\alpha}}\right) + \frac{\alpha(4+3\alpha)\gamma^2r^2-8(1+\alpha)}{(1+\alpha)^{3/2}\gamma r}\mathcal{K}_1\left(\frac{\gamma r}{\sqrt{1+\alpha}}\right)\right], \tag{35c}$$

$$E_{\frac{\sqrt{1+\alpha}}{\gamma}}(r) = \frac{1}{16\pi\alpha^2}\left\{-8\mathcal{K}_2(\gamma r) + \frac{8(1+\alpha)+\alpha^2\gamma^2r^2}{(1+\alpha)^2}\mathcal{K}_0\left(\frac{\gamma r}{\sqrt{1+\alpha}}\right) + \frac{4\left[4(1+\alpha)-\alpha\gamma^2r^2\right]}{(1+\alpha)^{3/2}\gamma r}\mathcal{K}_1\left(\frac{\gamma r}{\sqrt{1+\alpha}}\right)\right\}. \tag{35d}$$

This determines the compressible Brinkmanlet (29) completely. We next consider the behaviour as $r \to \infty$ in the direction $\boldsymbol{f}\cdot\boldsymbol{x} = 0$ perpendicular to the applied force. From the asymptotic behaviour as $r \to \infty$ of $\mathcal{K}_0, \mathcal{K}_1, \mathcal{K}_2$ [12], we find

$$\boldsymbol{u} \overset{\boldsymbol{f}\cdot\boldsymbol{x}=0}{\sim} \frac{\boldsymbol{f}}{\sqrt{8\pi}}\left\{e^{-\frac{\gamma r}{\sqrt{1+\alpha}}}\left[-\frac{(1+\alpha)^{7/4}r^{-3/2}}{\gamma^{3/2}(\gamma^2\varepsilon^2-1-\alpha)^2}+\cdots\right] + e^{-r/\varepsilon}\left[\frac{r^{1/2}}{2\varepsilon^{1/2}(\gamma^2\varepsilon^2-1)}+\cdots\right]\right\}, \tag{36}$$

where we have used the fact that $e^{-\gamma r} \ll e^{-\frac{\gamma r}{\sqrt{1+\alpha}}}$ for $\alpha > 0$. Hence there are two cases: if $\gamma\varepsilon < \sqrt{1+\alpha}$, then the first term dominates in the far-field and $\boldsymbol{u}\cdot\boldsymbol{f} < 0$. This is again the far-field signature of lateral vortices in the flow field. If however $\gamma\varepsilon > \sqrt{1+\alpha} > 1$, then the second term dominates, and $\boldsymbol{u}\cdot\boldsymbol{f} > 0$, which suggests that there are no lateral vortices

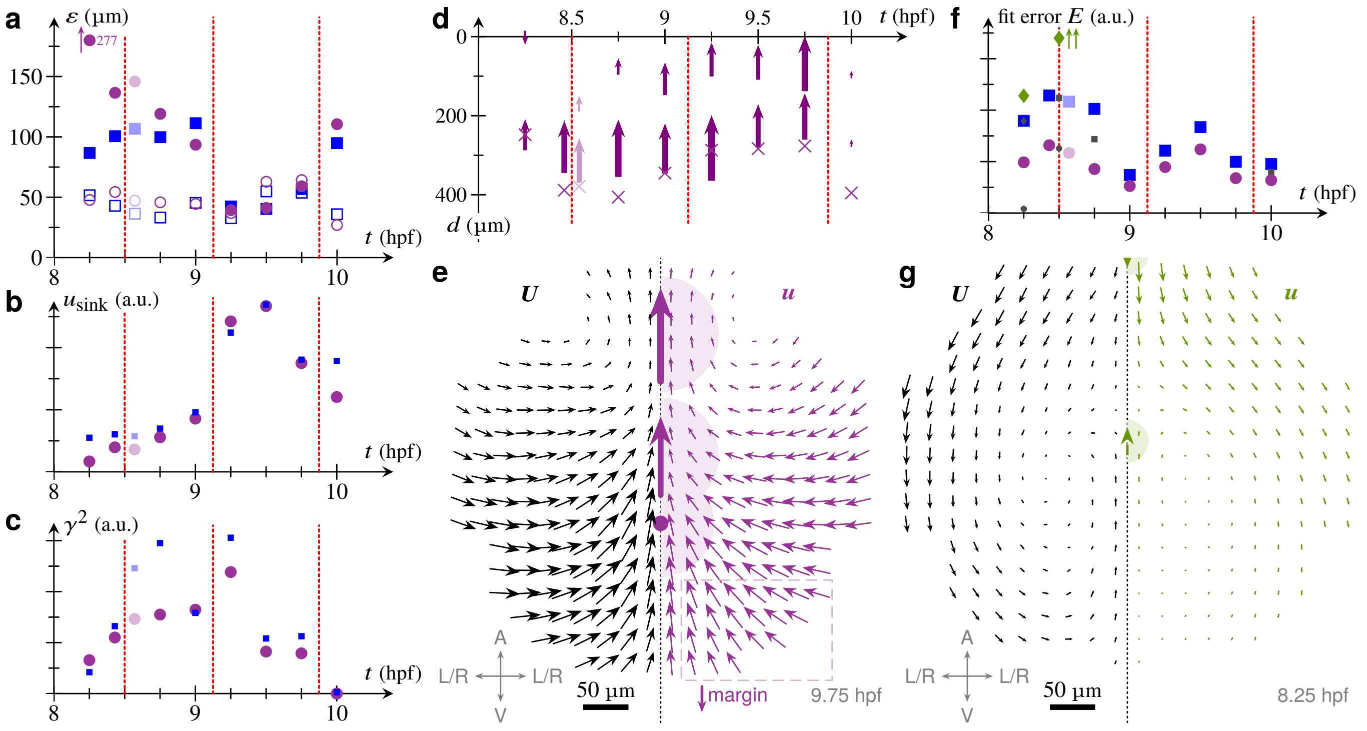

FIG. S1. Extensions of the model. (a) Fitted values of the sink regularisation radii ε_0 (filled markers) and force regularisation radii $\varepsilon_1 = \varepsilon_2$ (open markers), as a function of time t, expressed in hours-post-fertilisation (hpf). Results are shown for the model without margin (square markers) and the model with margin (round markers). Dashed vertical lines represent the three transitions discussed in the main text. Where two qualitatively different physical fits were found for one timepoint (close to one of these transitions), values for both fits are shown in a staggered fashion, with the temporal order decided by the other fits and the mechanical and biological interpretation of the transition. An offscale value is indicated by a vertical arrow. (b) Fitted fluid velocity u_{sink}, in arbitrary units (a.u.), induced by the sink at the position of the sink as a function of time t. Round markers show results for the model with margin; small square markers repeat the results for the model without margin from Fig. 4n of the main text for comparison. (c) Analogous plot of the fitted friction coefficient γ^2 against time t. Results for the model without margin are repeated from Fig. 4o of the main text. (d) Plot of the positions of the fitted regularised sinks (crosses) and of the positions, orientations, and magnitudes of the fitted regularised Brinkmanlets (arrows) as a function of time t, for the model with margin, analogous to Fig. 4m of the main text. The positions are expressed in terms of the distance d from the anterior end of the ANP. The image singuarities imposing the margin boundary condition are not shown. (e) Left-right symmetrised experimental flow field $\boldsymbol{U}$ (left), reproduced by the fitted flow field $\boldsymbol{u}$ (right) for the model with margin at $t = 9.75$ hpf, analogous to Fig. 4l of the main text. Arrows on the axis represent the positions, directions, and magnitudes of the fitted regularised Brinkmanlets; the dot represents the position of the fitted regularised Brinkman sinks. Shaded areas show the corresponding regularisation radii. The image singularities are, again, not shown. The dashed box highlights the underestimate of the left-right flows by the fit. The arrow and "margin" label emphasise the vegetal or posterior direction towards the boundary at which the margin boundary condition is applied. Inset: embryo axes (A: animal, V: vegetal; L/R: left-right symmetrised). (f) Fit error E, defined by Eq. (24), as a funtion of time t for the model without margin (square markers), the model with margin (round markers), and the infinitely compressible model (lozenge markers). Small markers show lower fit errors for discarded, unphysical fits for some timepoints. Double vertical arrows indicate an offscale value. (g) Experimental and fitted flow fields for the infinitely compressible model at $t = 8.25$ hpf, analogous to Fig. 4k of the main text.

in this case. This suggestion is confirmed by numerical calculations (not shown). This shows that, for the compressible Brinkmanlet, changes in ε can change the topology of the flow field; this is not the case for the incompressible Brinkmanlet discussed earlier, for which the far-field behaviour, expressed by Eq. (18), is independent of ε.

There is no analogous problem for a regularised sink flow; indeed, with $p = 0$ and $\boldsymbol{F} = \boldsymbol{0}$, the solution of Eq. (25a) decaying in the far-field is simply $\boldsymbol{u} = \boldsymbol{0}$. The mass conservation equation (25c) then constitutes a contradiction. This is the mathematical reason why we use a model of an incompressible fluid to represent the experimental flows and in particular the sink resulting from ingression.

C. Discussion

Results for these extensions of the model, as well as for the fitted regularisation radii not discussed in the main text, are shown in Fig. S1. We discuss these results and their physical and biological implications below.

1. *Fitted regularisation radii*

The fitted values of the regularisation radii allow for a consistency check of the fit: The force regularisation radii $\varepsilon_1 = \varepsilon_2$ represent the extent over which the mesendoderm exerts friction on the neuroectoderm, so are expected not to change over

time. Consistently with this expectation, the fitted values of $\varepsilon_1 = \varepsilon_2$ are roughly constant (Fig. S1a). By constrast, the fitted sink radius ε_0 decreases, as expected, as ingression movements become stronger (Fig. S1a): at this time, the sink represents localised ingression (small ε_0), but it represents the smeared-out, effective compressibility of the tissue (larger ε_0) at earlier times before ingression, due the assumption of incompressibility of the fluid. This interpretation is also consistent with the non-zero sink velocities fitted at the earliest timepoints (Fig. S1b), before the start of internalisation.

2. *The EVL/YSL margin*

By representing the tissue as an infinite two-dimensional fluid, the model neglects the boundary conditions at the EVL/YSL margin. Because the margin is closest to the ANP in the vegetal direction, we represent the EVL/YSL margin as a line boundary at $Y = Y_\mathrm{m} < 0$, at which we impose a boundary condition of no flow across the boundary, as described above. These boundary position and condition are, of course, still a simplification that neglects the flows arising from continued epiboly movements. In particular, this new boundary condition might be expected to strengthen left-right flows at the vegetal end of the ANP, which are underestimated by the model without this boundary condition (Fig. 4l in the main text). [This underestimate might also result, however and *a priori*, from the fact that the uncertainty in the experimental flow field (i.e. its left-right asymmetry) is larger there (not shown).] For fitting the model with this new boundary condition, we fix Y_m so that the boundary is approximately 800 µm behind the front of the ANP. This value corresponds to the margin position at 7.5 hpf. We make this choice to maximise the effect of the boundary condition even though, because of continued epiboly movements, the margin will be farther from the ANP at later stages.

The resulting fit (Figs. S1a–S1e) agrees with the fit without the margin. In particular, it recovers the three mechanical regimes discussed in the main text (Figs. S1b–S1d) and the fitted regularisation radii (Fig. S1a) continue to be consistent with expectations. Including the margin does not improve the fit of the vegetal left-right flows at late stages (Fig. S1e), though, suggesting that these discrepancies are due to the larger uncertainty of the experimental flows there. Finally, the fit error (Fig. S1f) decreases compared to the case without the margin (without additional fitting parameters, since Y_m is not fitted for). This suggests that the simplified margin boundary condition correctly captures additional physics of the system.

3. *Incompressibility*

As noted above, the assumption of an incompressible fluid implies that the compressibility of the tissue must be absorbed, at least for early stages, into an effective sink. An alternative description of the early stages assumes, as described earlier, an infinitely compressible fluid. Fitting only two regularised compressible Stokeslets representing the opposing forces from the mesendoderm, this model can represent the experimental flows at early stages quantitatively (Fig. S1g), but even at 8.25 hpf, the fit error is higher than that of the incompressible models with or without margin (Fig. S1f), and is much higher than that of the incompressible models from 8.5 hpf (Fig. S1f). (Note however that the compressible model has fewer fitting parameters.) This hints, physically, that a regularised sink in an incompressible fluid is a better mechanical representation of the compressibility of the system than an infinitely compressible fluid. It also hints, more biologically, that ingression movements (corresponding to a true sink, rather than an effective one) start to contribute to the experimental flows from 8.5 hpf, i.e. from the time of the first mechanical transition, when mesendoderm migration ceases to dominate the mechanics.

4. *Ingression as a sink*

We conclude by briefly discussing our modelling of ingression as a sink. We note that we rejected some fits as being unphysical, although such fits can be found at lower fit scores than the fits that we have retained (Fig. S1g). We declared some such fits to be unphysical because, for example, the fitted values of the regularisation radii were much larger than the physical extent of the neuroectoderm. The existence of such spurious fits is not surprising given the considerable nonlinearity of the fitting problem.

More interestingly, especially at late stages ($>$ 9 hpf), we found fits in which (almost) equal and opposite forces are nearly colocalised with the sink (not shown). While colocalisation of the two fitted forces contradicts our physical interpretation of these forces (as forces exerted on the neuroectoderm by the underlying mesendoderm), this may signify that the ingression flows are better represented by the superposition of a sink and a Brinkmanlet dipole. Fully understanding the physics of the ingression flows remains a question for future work. Meanwhile, extending our fitting procedure to fit two forces and such a superposition of a sink and a Brinkmanlet dipole is beyond the scope of our analysis, the more so as we cannot justify fitting four singularities in the same way as we justified the fitting of three singularities based on our argument showing that they are the minimal ingredients that can reproduce the flow fields even qualitatively (Figs. 4a–4j of the main text, and discussion thereof).

APPENDIX: INTEGRALS

In this Appendix, we give several integral identities required for the derivation of regularised singularities in this Supplemental Note. These identities can be obtained using Mathematica (Wolfram, Inc.).

Calculation of polar integrals in two-dimensional Fourier inversions requires the identities

$$\int_0^{2\pi} \mathrm{e}^{\mathrm{i}a\cos\theta}\,\mathrm{d}\theta = 2\pi\mathcal{J}_0(a), \tag{A1a}$$

$$\int_0^{2\pi} \mathrm{e}^{\mathrm{i}a\cos\theta}\cos\theta\,\mathrm{d}\theta = 2\mathrm{i}\pi\mathcal{J}_1(a), \tag{A1b}$$

$$\int_0^{2\pi} \mathrm{e}^{\mathrm{i}a\cos\theta}\sin^2\theta\,\mathrm{d}\theta = \frac{2\pi\mathcal{J}_1(a)}{a}, \tag{A1c}$$

valid for $a > 0$ and wherein $\mathcal{J}_0, \mathcal{J}_1, \dots$ again denote the Bessel functions of the first kind.

The calculations in this Supplemental Note further rely on the identities

$$\int_0^\infty \frac{\ell \mathcal{J}_0(a\ell)}{1+\ell^2}\,\mathrm{d}\ell = \mathcal{K}_0(a), \tag{A2a}$$

$$\int_0^\infty \frac{\mathcal{J}_1(a\ell)}{1+\ell^2}\,\mathrm{d}\ell = \frac{1}{a} - \mathcal{K}_1(a), \tag{A2b}$$

$$\int_0^\infty \frac{\ell \mathcal{J}_0(a\ell)}{\left(1+\ell^2\right)^2}\,\mathrm{d}\ell = \frac{a\mathcal{K}_1(a)}{2}, \tag{A2c}$$

$$\int_0^\infty \frac{\mathcal{J}_1(a\ell)}{\left(1+\ell^2\right)^2}\,\mathrm{d}\ell = \frac{1}{a} - \frac{a\mathcal{K}_2(a)}{2}, \tag{A2d}$$

valid again for $a > 0$, and in which $\mathcal{K}_0, \mathcal{K}_1, \mathcal{K}_2, \dots$ denote again the modified Bessel functions of the second kind.

[1] Cæsar, *Commentarii de bello Gallico* (Ancient Rome); for a modern translation, see, e.g., *The Gallic War* (Penguin Classics, London, UK, 1982).

[2] C. Pozrikidis, *Introduction to Theoretical and Computational Fluid Dynamics*, 2nd ed. (Oxford University Press, New York, NY, 2011) Chaps. 2.1, 6.1, 6.5, 6.6, pp. 112–123, 370–381, 410–431.

[3] M. A. Goldshtik, Viscous-flow paradoxes, Ann. Rev. Fluid Mech. **22**, 441 (1990).

[4] More mathematically, these anterior or posterior stagnation points disappear if Stokes' paradox is solved by the addition of a friction force $-\gamma^2\boldsymbol{u}$ to the flow as in Sec. II. Indeed, taking the limit $\varepsilon \to 0$ in Eqs. (16) using Mathematica, the flow along the axis defined by $\boldsymbol{f}$ is, from Eq. (11), $\boldsymbol{u} = [A_0(r) + B_0(r)]\boldsymbol{f}$, with $A_0(r) + B_0(r) = [1 - \gamma r\mathcal{K}_1(\gamma r)]/(2\pi r^2\gamma^2) > 0$, since $x\mathcal{K}_1(x) < 1$ for $x > 0$. This shows, mathematically, that there are no stagnation points on the flow axis.

[5] H. C. Brinkman, A calculation of the viscous force exerted by a flowing fluid on a dense swarm of particles, Flow Turb. Combust. **1**, 27 (1949).

[6] R. Cortez, The method of regularized Stokeslets, SIAM J. Sci. Comput. **23**, 1204 (2001).

[7] R. Cortez, L. Fauci, and A. Medovikov, The method of regularized Stokeslets in three dimensions: Analysis, validation, and application to helical swimming, Phys. Fluids **17**, 031504 (2005).

[8] D. J. Smith, A nearest-neighbour discretisation of the regularized stokeslet boundary integral equation, J. Comp. Phys. **358**, 88 (2018).

[9] C. Pozrikidis, *Boundary Integral and Singularity Methods for Linearized Viscous Flow*, Cambridge Texts in Applied Mathematics (Cambridge University Press, Cambridge, UK, 1992).

[10] B. Zhao, E. Lauga, and L. Koens, Method of regularized stokeslets: Flow analysis and improvement of convergence, Phys. Rev. Fluids **4**, 084104 (2019).

[11] K. Leiderman and S. D. Olson, Swimming in a two-dimensional Brinkman fluid: Computational modeling and regularized solutions, Phys. Fluids **28**, 021902 (2016).

[12] M. Abramowitz and I. A. Stegun, *Handbook of Mathematical Functions*, 10th ed., Applied Mathematics Series, Vol. 55 (National Bureau of Standards, 1972) Chap. 9, pp. 355–389.

[13] Throughout this Supplemental Note, we adopt the convention that the Fourier transform $\tilde{f}$ of a suitable function f on $\mathbb{R}^2$ is

$$\tilde{f}(\boldsymbol{k}) = \iint_{\mathbb{R}^2} f(\boldsymbol{x})\mathrm{e}^{-\mathrm{i}\boldsymbol{k}\cdot\boldsymbol{x}}\,\mathrm{d}^2\boldsymbol{x}.$$

With this convention,

$$f(\boldsymbol{x}) = \frac{1}{(2\pi)^2}\iint_{\mathbb{R}^2} \tilde{f}(\boldsymbol{k})\mathrm{e}^{\mathrm{i}\boldsymbol{k}\cdot\boldsymbol{x}}\,\mathrm{d}^2\boldsymbol{k}$$

is the form taken by the Fourier inversion theorem in two dimensions.

[14] Mathematica (Wolfram, Inc.) was used for the calculations in the Supplemental Note to assist with manipulating complicated algebraic expressions.

[15] In more detail, we fit the experimental data in two steps: first, for each stage, we fit $y_0, y_1, y_2, q, f_1, f_2$ at fixed values of $\gamma, \varepsilon_0, \varepsilon_1 = \varepsilon_2$. At this stage, we also fit separately for different signs of f_1, f_2, and different relative positions of the singularities. We then seek local minima of E on the grid of $\gamma, \varepsilon_0, \varepsilon_1 = \varepsilon_2$, and use these as initial guesses for a second fitting round with respect to all parameters. This enables us to find local minima in the fitting landscape, and hence discard unphysical fits resulting from unphysical global minima for some timepoints (as discussed further in Sec. III C of this Supplemental Note).

[16] D. J. Acheson, *Elementary Fluid Dynamics*, Oxford Applied Mathematics and Computer Sciences Series (Clarendon Press, Oxford, UK, 2009) Chap. 6.3, pp. 205–207.

[17] I. G. Currie, *Fundamental Mechanics of Fluids*, 2nd ed. (McGraw-Hill, New York, NY, 1993) Chaps. 1.11, 1.12, 1.13, pp. 23–29.

[18] G. K. Batchelor, *An Introduction to Fluid Dynamics*, Cambridge Mathematical Library (Cambridge University Press, Cambridge, UK, 2000) Chap. 3.1, pp. 131–137.

[19] For a one-dimensional model of a biological tissue, P. Recho, J. Ranft, and P. Marcq, One-dimensional collective migration of a proliferating cell monolayer, Soft Matter **12**, 2381 (2016) obtain $P(\rho) \sim -P_0 \log(\rho/\rho_0)$, where $P_0 = \ell/\tau$, with ℓ an Onsager coefficient and τ the characteristic time scale of cell division. In particular, $p \approx 0$ holds for very slow cell divisions.

[20] M. Smutny, Z. Ákos, S. Grigolon, S. Shamipour, V. Ruprecht, D. Čapek, M. Behrndt, E. Papusheva, M. Tada, B. Hof, T. Vicsek, G. Salbreux, and C.-P. Heisenberg, Friction forces position the neural anlage, Nat. Cell Biol. **19**, 306 (2017).
