# Supplementary material for: A multi-tiered mechanical mechanism shapes the early neural plate
Source: Nat Commun. 2025 Jul 4;16:6187. doi: 10.1038/s41467-025-61303-1 (PMC12227548; doi:10.1038/s41467-025-61303-1)
Supplement: Supplementary file 2 — Description of Additional Supplementary Information [file 41467_2025_61303_MOESM2_ESM.pdf]

Description of Additional Supplementary Files

File Name: Supplementary Movie 1
Description: Live imaging of anterior neural plate dynamics in wild type embryo. Timelapse imaging of Tg(otx2:Venus) embryo injected with membrane RFP (mRFP) mRNA from 7.5 to 10 hpf. Neuroectoderm cells (green) and cell outlines (magenta). Animal pole view; anterior up, posterior down. Time, minutes.

File Name: Supplementary Movie 2
Description: Live imaging of neuroectoderm internalisation in wild type embryo. Time-lapse imaging of Tg(otx2:Venus) embryo injected with membrane RFP (mRFP) mRNA from 7.5 to 10 hpf. Neuroectoderm cells (green) and cell outlines (magenta). Sagittal section; anterior left, posterior right; dorsal up, ventral down. Time, minutes.

File Name: Supplementary Movie 3
Description: Live imaging of mesendoderm actin dynamics in wild type embryo. Time-lapse imaging of Tg(actb2:Lifeact-GFP) embryo at 8 hpf. Left/right movie shows mesendoderm cells at the front/rear of the collective, respectively. Section of animal pole/dorsal view; anterior left, posterior right; right up, left down. Fire LUT, arbitrary units, 0 (dark blue) – 256 (white). Scale bar, 20µm. Time, minutes.

File Name: Supplementary Movie 4
Description: Live imaging of neuroectoderm internalisation in cdh1 morphant embryo. Time-lapse imaging of Tg(otx2:Venus) embryo injected with cdh1 morpholino and membrane RFP (mRFP) mRNA from 7.5 to 9.5 hpf. Neuroectoderm cells (green) and cell outlines (magenta). Sagittal section; anterior left, posterior right; dorsal up, ventral down. Time, minutes.

File Name: Supplementary Movie 5
Description: Live imaging of anterior neural plate dynamics in lefty1 morphant embryo. Time-lapse imaging of Tg(otx2:Venus) embryo injected with lefty 1 morpholino and membrane RFP (mRFP) mRNA from 7.5 to 10 hpf. Neuroectoderm cells (green) and cell outlines (magenta). Animal pole view; anterior up, posterior down. Time, minutes.

File Name: Supplementary Movie 6
Description: Live imaging of neuroectoderm internalisation in wnt11 mutant embryo. Time-lapse imaging of Tg(otx2:Venus); wnt 11 mutant embryo injected with membrane RFP (mRFP) mRNA from 7.5 to 9.5 hpf. Neuroectoderm cells (green) and cell outlines (magenta). Sagittal section; anterior left, posterior right; dorsal up, ventral down.
